# Enabling Quantitative Benchtop ^13^C NMR Spectroscopy in Fast Continuous Flow

**DOI:** 10.1002/mrc.70119

**Published:** 2026-05-15

**Authors:** Sarah Mross, Hans Hasse, Kerstin Münnemann

**Affiliations:** ^1^ Laboratory of Engineering Thermodynamics (LTD) RPTU Kaiserslautern Germany; ^2^ Laboratory of Advanced Spin Engineering ‐ Magnetic Resonance (LASE‐MR) RPTU Kaiserslautern Germany

## Abstract

Continuous‐flow benchtop NMR spectroscopy is highly attractive for quantitative reaction and process monitoring. Although 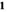
H NMR spectroscopy is widely used, signal overlap frequently compromises quantitative analysis. In principle, this limitation can be addressed by 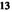
C NMR spectroscopy; however, its application is restricted by the inherently low sensitivity of 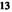
C nuclei. This limitation is further aggravated by insufficient polarization buildup in benchtop NMR spectroscopy in fast continuous flow, as the compact design provides only short residence times in the premagnetization zone. We demonstrate here that the latter limitation can be overcome by paramagnetic relaxation enhancement (PRE). Furthermore, we demonstrate that applying PRE is particularly effective when combined with a method that enhances weak 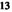
C polarization, such as 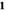
H to 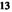
C polarization transfer. For this, we used PENDANT. We report the first application of PRE (with and without PENDANT) for quantitative analysis of mixtures with continuous‐flow 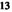
C benchtop NMR spectroscopy. Experiments were carried out for common solvents (acetonitrile, 1,4‐dioxane, and ethanol) as well as binary and ternary mixtures of these solvents over a wide range of flow rates. The results demonstrate that PRE, especially when combined with PENDANT, enables robust quantitative analysis with 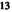
C NMR spectroscopy even at high flow rates, significantly expanding the applicability of benchtop NMR instruments for quantitative reaction and process monitoring.

## Graphical abstract

Paramagnetic relaxation enhancement (PRE) accelerates polarization buildup in continuous‐flow benchtop C NMR spectroscopy by the use of an immobilized PRE agent. By combining PRE with PENDANT polarization transfer, weak C signals are enhanced even at high flow rates, enabling robust quantitative analysis of solvent mixtures under process‐relevant flow conditions.

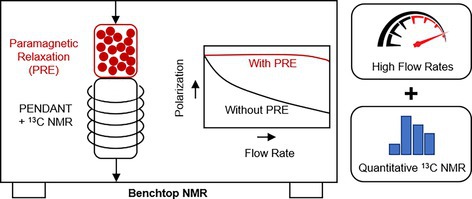

## 1 Introduction

Nuclear magnetic resonance (NMR) spectroscopy is a powerful technique for reaction and process monitoring [1, 2, 3]. Benchtop NMR spectrometers are particularly well suited to these applications owing to their compact design, which enables installation in close proximity to the monitored process [3, 4, 5, 6]. In many such applications, measurements must be performed on flowing samples.

Due to its high sensitivity, 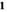
H NMR is generally preferred for benchtop flow applications; however, the limited chemical‐shift dispersion of protons often results in severe signal overlap in multicomponent mixtures, which restricts its usefulness for quantitative reaction and process monitoring. 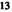
C NMR spectroscopy, by contrast, offers substantially improved spectral resolution but suffers from inherently lower sensitivity. In continuous‐flow experiments with benchtop instruments, insufficient premagnetization further aggravates this limitation, as short residence times in the small premagnetization zone of the compact instruments restrict polarization buildup and thus reduce signal intensities [4, 7].

With increasing flow velocity, the relative polarization ( S/ $S_{0}$, where S is the signal integral under flow and $S_{0}$ is the corresponding static signal integral) decreases exponentially as a function of the spin‐lattice relaxation time $T_{1}$, and the residence time τ in the premagnetization zone, as described by Equation (1) [8, 9].

(1) $$\frac{S}{S_{0}}\;=\;1\;-\;\exp\left(-\frac{\tau }{T_{1}}\right)\;\;\;\;\;\;\;\;\;\;\text{with}\;\;\tau =\frac{L_{\text{pol}}}{\nu }$$

The residence time τ can be calculated from the polarization length $L_{\text{pol}}$ and the flow velocity ν, see Equation (1), where $L_{\text{pol}}$ is determined by the spectrometer design, in particular the magnet size, and is significantly shorter in compact benchtop instruments than in high‐field NMR systems [10]. The $T_{1}$ time depends on the nucleus and its molecular environment, including the solvent. The resulting loss of polarization restricts the range of accessible flow velocities in benchtop NMR experiments [4, 11], an effect that is particularly pronounced for nuclei with long $T_{1}$ times, such as 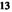
C. Together with the inherently low sensitivity of the 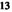
C nucleus, this renders quantitative measurements under continuous‐flow conditions with benchtop NMR instruments impractical for many applications, as, for example, fast reactions.

Hyperpolarization techniques such as Overhauser dynamic nuclear polarization (ODNP) [12, 13, 14, 15, 16, 17] and signal amplification by reversible exchange (SABRE) [18, 19, 20] or polarization‐transfer approaches [15, 21, 22, 23] can, in principle, be employed to overcome the signal‐to‐noise ratio (SNR) limitations. ODNP and SABRE, however, require specialized instrumentation and experimental setups [14, 15]. An alternative and considerably simpler strategy is the use of paramagnetic relaxation enhancement (PRE) [24, 25, 26, 27]. In this approach, a PRE agent [10, 14, 28, 29, 30, 31, 32, 33] is incorporated as a fixed bed within the premagnetization zone of the benchtop NMR spectrometer. Upon passing through the fixed bed, polarization is rapidly built up in the liquid due to substantially reduced $T_{1}$ times, leading to high signal intensities in the detection [34] even at elevated flow rates. Comparable polarization enhancement could, in principle, also be achieved using homogeneous dissolved PRE agents; however, their presence in the detection volume could result in severe line broadening, including sample contamination, and the inability to reuse the PRE agent. For this reason, heterogeneous PRE agents are required in practice.

The key advantages of PRE for benchtop NMR spectroscopy under continuous‐flow conditions have already been demonstrated in earlier work from our group [24] for quantitative 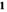
H NMR measurements. In the present study, we show that, when applied to 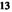
C NMR spectroscopy, PRE becomes an enabling technology, opening a practical route to exploiting this powerful method for reaction and process monitoring.

The use of PRE alone, however, cannot overcome the inherently low sensitivity of 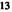
C NMR spectroscopy, which arises from the low gyromagnetic ratio and low natural abundance of 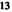
C [10, 21]. Consequently, even under conditions of full premagnetization, quantitative analysis would require either a large number of scans or the use of isotopically enriched compounds — both of which are generally impractical for reaction and process monitoring. This limitation can be addressed by applying ^1^H to 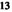
C polarization‐transfer techniques, in particular PENDANT (abbreviation for “polarization enhancement that is nurtured during attached nucleus testing”) [35, 36], which combine the high sensitivity of 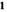
H NMR with the large chemical‐shift dispersion of 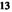
C NMR. Here, we demonstrate that PENDANT can be combined with the rapid polarization buildup induced by PRE agents, enabling quantitative analysis of complex, fast‐flowing mixtures by benchtop 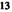
C NMR spectroscopy without isotope enrichment and with only a small number of scans.

Previous studies have reported the use of immobilized PRE agents in fixed beds for 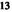
C NMR measurements of flowing mixtures in high‐field NMR spectrometers [26, 27]. While the authors achieved substantial improvements in SNR, they did not obtain quantitative results. The novelty of the present work is therefore threefold: First, we demonstrate that PRE can be used to overcome the premagnetization limitations inherent to benchtop 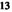
C NMR spectroscopy; second, we show that quantitative results can be obtained from PRE‐enhanced continuous‐flow 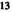
C NMR spectroscopy; and third, we demonstrate that further substantial gains are achieved by combining PRE with the polarization‐transfer technique PENDANT. In summary, this work opens a practical route to quantitative reaction and process monitoring in fast continuous‐flow using 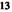
C benchtop NMR spectroscopy.

## 2 Experimental Section

### 2.1 Chemicals and Sample Preparation

Acetonitrile (ACN, ≥ 99.9%), 1,4‐dioxane (DIOX, ≥ 99.8%), and ethanol (EOH, ≥ 99.9%) were purchased from Sigma‐Aldrich. All chemicals were used as received. Stock solutions (80 g) of the binary and ternary mixtures were prepared gravimetrically using a laboratory balance (XS603S DeltaRange, Mettler Toledo) with an accuracy of ± 10 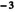 g g 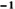. The composition of the stock solutions is given in Table 1.

**TABLE 1:** Composition of the studied binary and ternary mixtures.

System	Mixture	Component i	$x_{i}$ (mol mol 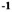 )
1: ACN + EOH	1	ACN	0.75
	2		0.50
	3		0.25
2: ACN + DIOX	4	ACN	0.75
	5		0.50
	6		0.25
3: ACN + EOH + DIOX	7	ACN	0.60
		EOH	0.20
	8	ACN	0.33
		EOH	0.33
	9	ACN	0.20
		EOH	0.20
	10	ACN	0.20
		EOH	0.60

### 2.2 Experimental Setup

**FIGURE 1:**
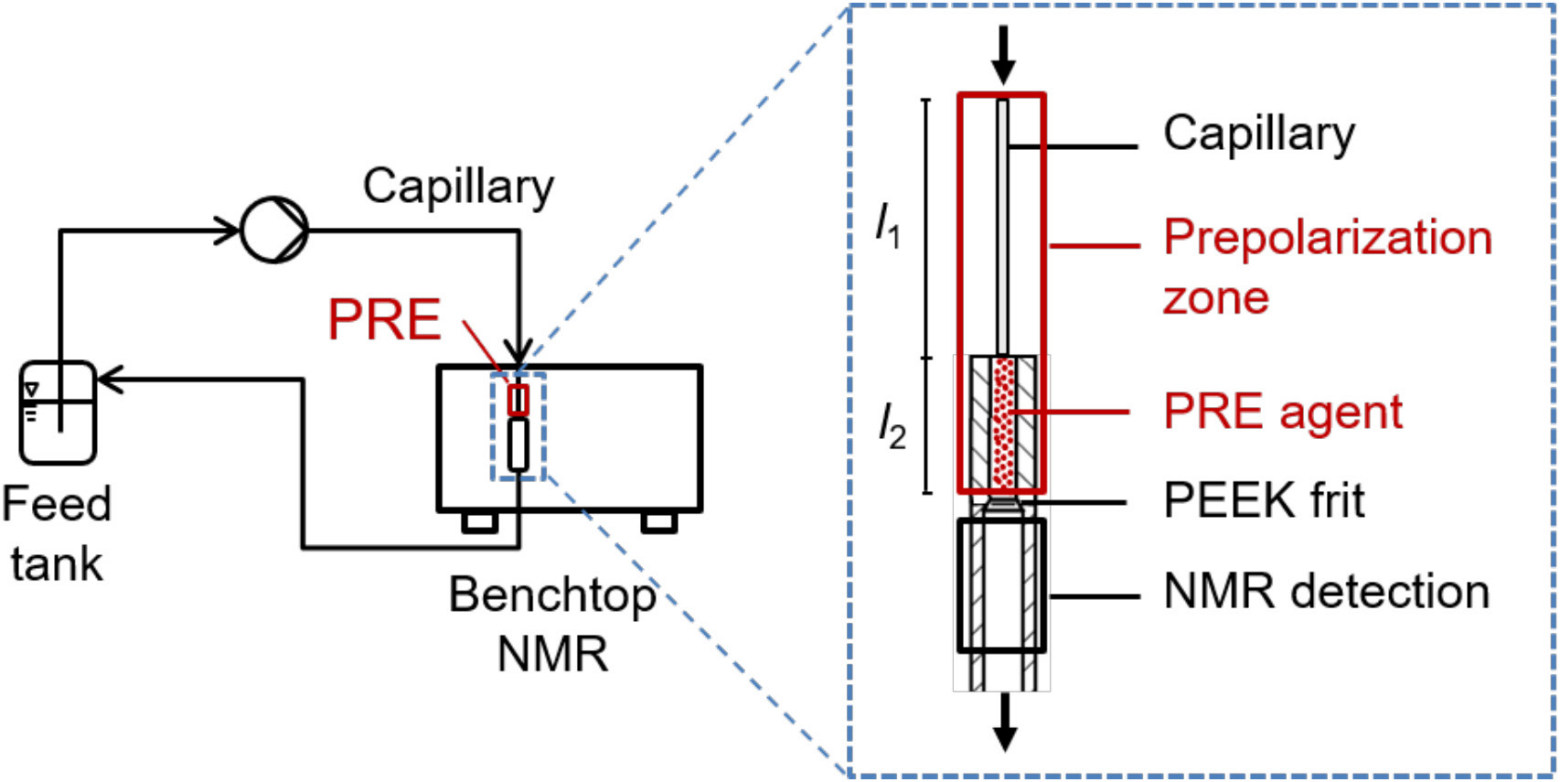
Experimental setup for 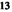
C continuous‐flow benchtop NMR with PRE. The premagnetization zone consists of the PEEK capillary (length $l_{1}$) and the PRE agent cell ( $l_{2}$).

The experimental setup (Figure 1) was similar to that used in our previous work [24]. The feed was supplied continuously from a tank to the NMR instrument through a PEEK capillary (inner diameter, d=1 mm) using a high‐pressure pump (WADose Plus HP, Flusys). The pump was calibrated for volumetric flow rates $\dot{V}$ between 0.5 and 5.0 ml min 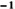. Prior to entering the detection cell, the feed passed through a fixed bed filled with the PRE agent, identical to that used in our earlier study [24]: nitroxide radicals (4‐glycidyloxy‐2,2,6,6‐tetramethylpiperidine‐1‐oxyl, GTEMPO, 120 mM) immobilized via a polymeric linker on controlled porous glass beads. As reported in our previous studies [14, 24], the PRE agent exhibits high stability in different solvents and under acidic conditions, both under stagnant and continuous‐flow operation.

PEEK containers were used to construct both the detection cell ( d=3 mm, length $l_{3}=15$ mm) and the fixed bed cell ( d=2 mm, $l_{2}=17$ mm). In this setup, the premagnetization zone consists of the capillary ( $l_{1}=83$ mm) and the PRE agent (fixed bed) cell. This corresponds to the prepolarization length $L_{\text{POL}}$ of 100 mm of the benchtop NMR spectrometer that was used in this work. The fixed bed was mounted directly upstream of the detection cell inside the bore of the benchtop NMR spectrometer and was equipped with a PEEK frit (pore size 2 μm, UP A‐700, Upchurch Scientific) to retain the PRE agent. Reference experiments were conducted using the same flow configuration but without the PRE agent. Details about flow velocities and residence times for this setup with and without the PRE agent are given in the Supporting Information.

### 2.3 NMR Measurements

A 42.5 MHz benchtop NMR spectrometer from Magritek (Spinsolve Carbon) was used. Quantitative 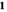
H and 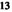
C NMR measurements were carried out using the Spinsolve Expert software (Magritek) with the following settings: 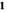
H NMR measurements in continuous flow were carried out with an excitation pulse angle of 90°, 3.5 to 12.5 s relaxation delay (depending on the flow rate), 1.6 s acquisition time, zero filling factor of 1, and one scan. For stop 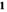
H NMR measurements (reference for the calculation of relative polarization in continuous flow), the relaxation delay was set to 25 s and the acquisition time to 3.2 s. 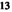
C NMR measurements in continuous‐flow were carried out using 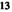
C inverse gating and 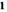
H decoupling with 3.5 to 12.5 s relaxation delay (depending on the flow rate), 1.6 s acquisition time, zero filling factor of 4, and 64 scans. For stop 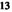
C NMR measurements, the relaxation delay was set to 150 s. PENDANT + 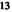
C NMR measurements were carried out using the implemented pulse sequence from our previous works [21, 37, 38] with 3.5 to 12.5 s relaxation delay (depending on the flow rate), 140 Hz for the coupling constant, 1.6 s acquisition time, zero filling factor of 4, and 8‐64 scans in continuous flow. For stop PENDANT + 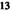
C NMR measurements, the relaxation delay was set to 25 s. Baseline correction, phase correction and apodization (1 Hz, only for 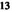
C) of all spectra were performed manually with MNova (Mestrelab Research).

The results of 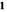
H NMR spectroscopy (stop and continuous flow) are an average of 10 individual measurements, and the results of 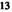
C NMR and PENDANT + 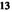
C NMR spectroscopy are an average of five individual measurements. The mole fraction of component i was calculated using Equation (2), where $A_{i}$ is the normalized peak area of component i.

(2) $$x_{i}=\frac{A_{i}}{\overset{N}{\underset{i=1}{\sum }}A_{i}}$$

The experimental uncertainty $\Delta x_{i}$ was determined by applying the Gaussian error propagation law to Equation (2), where the uncertainty $\delta A_{i}$ is the standard deviation of the averaged $A_{i}$.

To enable quantitative analysis of the PENDANT experiments, a calibration procedure is required. In PENDANT, the efficiency of polarization transfer depends on the local environment and thus varies between different functional groups. As a consequence, signal intensities in PENDANT‐enhanced 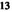
C NMR spectra are not directly proportional to the number of contributing nuclei under otherwise identical acquisition conditions.

To account for these group‐specific enhancement effects, reference measurements were performed for each mixture using conventional 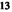
C NMR and PENDANT‐enhanced 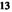
C NMR under static conditions in a 5 mm NMR tube, with 64 scans in each case. From these measurements, an enhancement factor ε was determined according to Equation (3): 

(3)

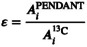

where $A_{i}^{\text{PENDANT}}$ denotes the normalized peak area of component i obtained from the PENDANT‐enhanced 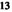
C NMR spectrum and 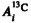 is the corresponding normalized peak area from the conventional 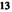
C NMR spectrum. The resulting enhancement factors were subsequently used to correct the signal intensities in the continuous‐flow PENDANT + 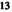
C NMR measurements, enabling quantitative evaluation of the experimental data.

## 3 Results and Discussion

### 3.1 Reduction of Relaxation Time $T_{1}$ by PRE Agent

Table 2 gives the relaxation times $T_{1}^{0}$ and $T_{1}^{\text{PRE}}$ in absence of the PRE agent and with the PRE agent, respectively, at 42.5 MHz for 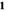
H and 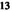
C nuclei as well as the efficiency E of the PRE agent in $T_{1}$ reduction. In all cases, E is at least 95%, highlighting the substantial reduction of $T_{1}$. Measurement details are provided in the Supporting Information, where also measured 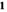
H $T_{1}^{0}$ times of all studied mixtures are reported.

**TABLE 2:** Relaxation times of the pure solvents measured at 42.5 MHz without ( $T_{1}^{0}$) and with the PRE agent ( $T_{1}^{\text{PRE}}$) and the corresponding PRE efficiency E.

Nucleus	Solvent	$T_{1}^{0}$ (s)	$T_{1}^{\text{PRE}}$ (s)	E (%)
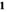 H	ACN ( ${\text{CH}}_{3}$) [24]	3.99 ( ±0.00)	0.05 ( ±0.00)	98
	DIOX ( ${\text{CH}}_{2}$)	2.78 ( ±0.00)	0.12 ( ±0.00)	96
	EOH ( ${\text{CH}}_{3}$) [24]	2.36 ( ±0.01)	0.06 ( ±0.00)	97
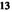 C	ACN ( ${\text{CH}}_{3}$)	15.41 ( ±0.24)	0.34 ( ±0.01)	98
	DIOX ( ${\text{CH}}_{2}$)	10.24 ( ±0.04)	0.54 ( ±0.08)	95
	EOH ( ${\text{CH}}_{3}$)	7.30 ( ±0.34)	0.35 ( ±0.04)	95

**FIGURE 2:**
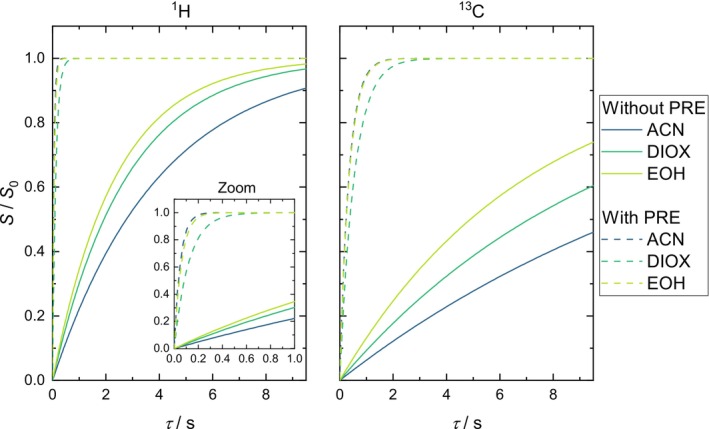
Relative polarization $S/S_{0}$ in continuous‐flow NMR as a function of the residence time τ in the premagnetization volume calculated with Equation (1) and the $T_{1}$ times from Table 2 for 42.5 MHz in this setup. Results for 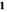
H (left) and 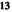
C (right) in different solvents with (dashed) and without (straight) PRE agent are shown.

The influence of $T_{1}$ on the relative polarization of the pure solvents is shown in Figure 2 for 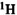 and 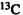 nuclei for different residence times τ (corresponding to the investigated flow rates between 0.5 and 5.0 mL min 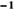) in the premagnetization zone of this setup. For the calculation of τ, the premagnetization volume was calculated assuming an ideal dense packing of the fixed bed with a void fraction of 0.26 (for more details, see Supporting Information). The relative polarization was calculated using Equation (1) and the data on $T_{1}^{0}$ and $T_{1}^{\text{PRE}}$ from Table 2.

As the $T_{1}$ time and flow rate increase (i.e., as the residence time τ decreases), the relative polarization decreases due to incomplete polarization buildup. For 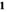
H nuclei, the relative polarization is higher compared with the 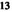
C nuclei because of the shorter $T_{1}$ times. Furthermore, it can be seen that, for this setup and the investigated residence times, complete polarization neither of 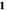
H or 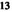
C nuclei can be achieved for the studied solvents. When the relative polarization is calculated using the $T_{1}^{\text{PRE}}$ times in contact with the PRE agent, there is nearly no loss of relative polarization for 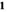
H for the investigated residence times. For 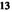
C, the polarization buildup is enhanced, showing only a decrease in relative polarization for residence times shorter than 2 s. The PRE agent enables complete polarization buildup for both 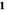
H and 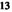
C nuclei in this setup for the majority of residence times.

### 3.2 System 1: Acetonitrile + Ethanol

We discuss here only results for 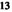
C NMR. Additional results from experiments in which the application of PRE for 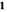
H was studied are presented in the Supporting Information. For simplicity, all results in the following are plotted as a function of flow rate $\dot{V}$ instead of residence time, because τ differs for measurements with and without (reference) the PRE agent, see the Supporting Information.

#### 3.2.1 C NMR Spectra

**FIGURE 3:**
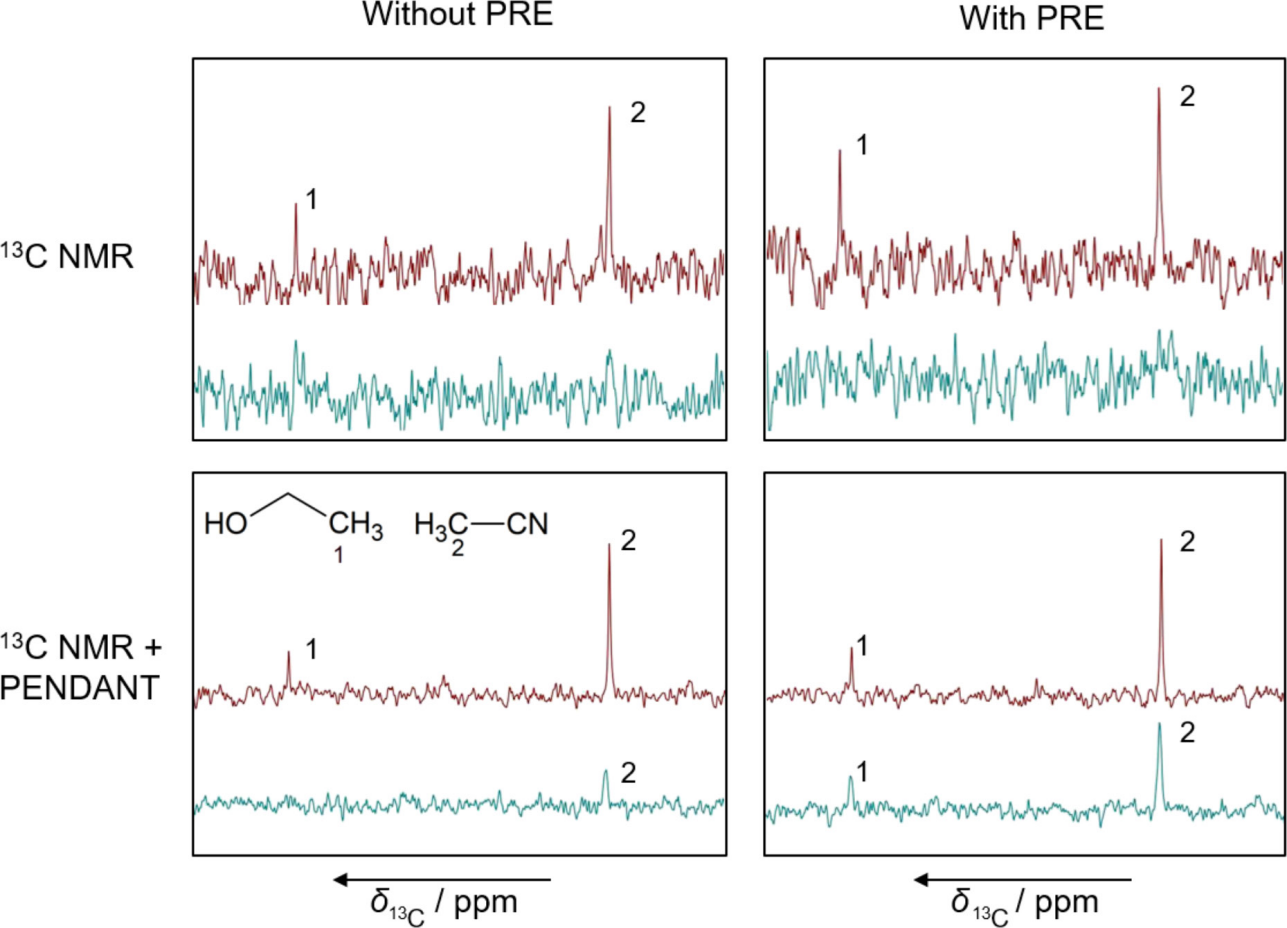
Continuous‐flow 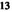
C NMR spectra of acetonitrile (ACN) + ethanol (EOH) (Mixture 1, Table 1). The spectra were acquired at two flow rates, 0.5 (red) and 5.0 ml ${\text{min}}^{-1}$ (turquoise). The spectra acquired without PENDANT (upper panels) were acquired using 64 scans, those acquired with PENDANT (lower panels) using eight scans. Panels on the left were recorded without PRE and panels on the right with PRE.

Figure 3 shows eight continuous‐flow benchtop 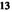
C NMR spectra of Mixture 1 (ACN + EOH, cf. Table 1), arranged in four panels. Each panel contains two spectra acquired at different flow rates. Owing to the reduced residence time in the premagnetization zone, the signal intensity is consistently lower at the high flow rate (turquoise) than at the low flow rate (red). The upper two panels display 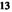
C NMR spectra acquired without PENDANT using 64 scans, whereas the lower two panels show spectra acquired with PENDANT using only eight scans. Despite this substantial reduction in acquisition time, the SNR is clearly improved when using PENDANT. The greatest benefit from applying PRE is expected for high flow rates (turquoise). However, without PENDANT (upper panels), no useful 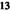
C NMR spectra were obtained at high flow rates (turquoise) — even when PRE was applied, as no EOH signal could be detected. In contrast, combining PENDANT (lower panels) with PRE (right), yielded quantifiable spectra, even at the high flow rates (turquoise).

#### 3.2.2 Prepolarization Efficiency

**FIGURE 4:**
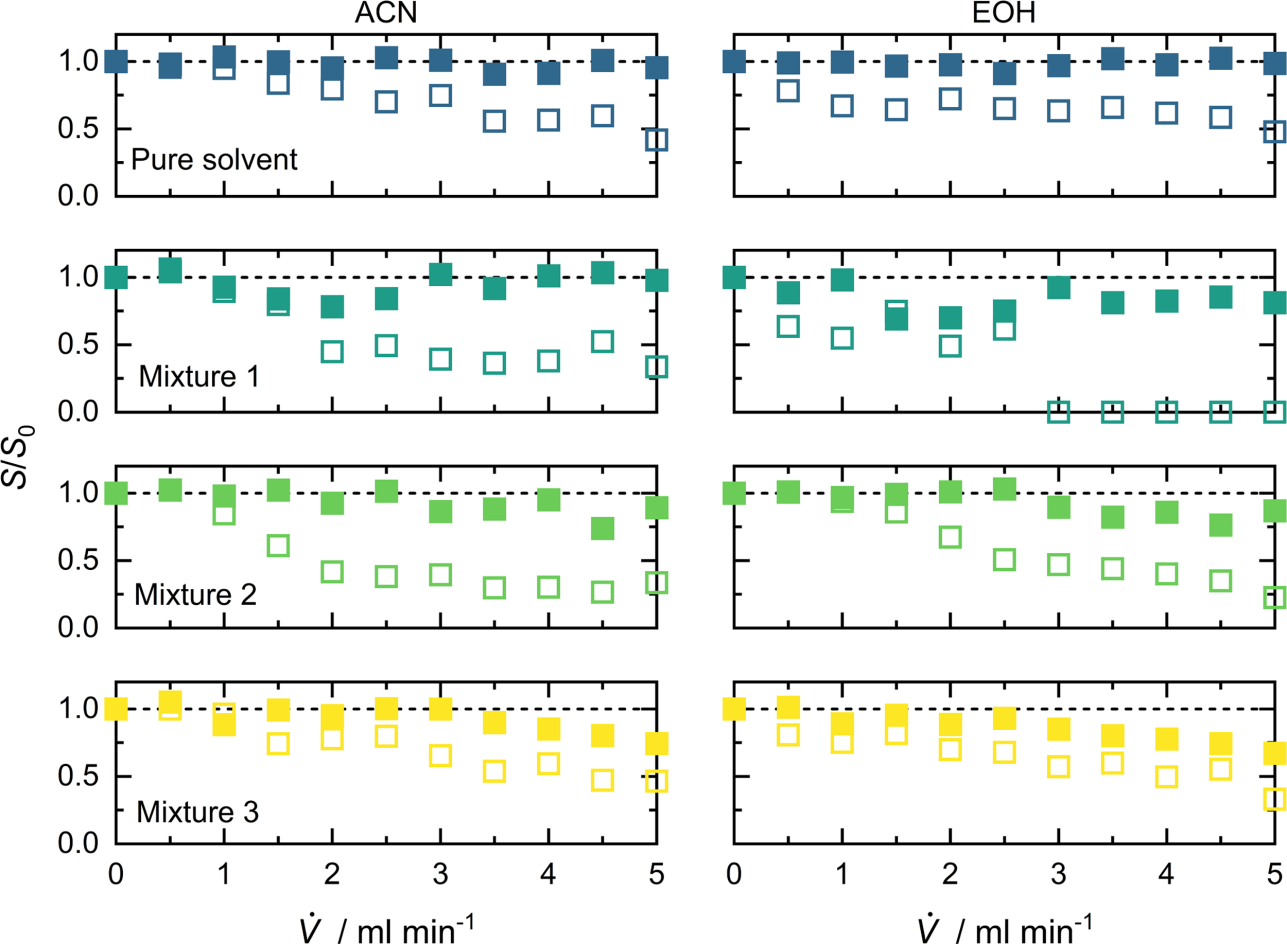
Relative polarization S/ $S_{0}$ of acetonitrile (ACN, left) and ethanol (EOH, right) for the pure solvents and the studied ACN + EOH mixtures (Mixtures 1–3, Table 1) as a function of flow rate. Spectra were recorded using PENDANT with eight scans. Data obtained with PRE are shown as filled symbols, data obtained without PRE as open symbols; dashed lines indicate the static reference experiments. Error bars are within symbol size.

Figure 4 shows the relative polarization S/ $S_{0}$ of ACN (left) and EOH (right) for the pure solvents and the three investigated ACN + EOH binary mixtures (Mixtures 1–3; see Table 1) as a function of flow rate. The corresponding numerical data are given in the Supporting Information. All spectra were recorded using PENDANT with eight scans. Results obtained with PRE (filled symbols) are compared with measurements without PRE (open symbols) and to the static reference experiments (dashed line). With PRE, full premagnetization is achieved in most cases; only at high flow rates a reduction in relative polarization is observed in some instances. In contrast, experiments performed without PRE show a pronounced decrease in relative polarization across all studied systems. In Mixture 1 ( $x_{\text{ACN}}=0.75$ mol mol 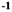), the EOH signal was indistinguishable from the baseline noise at the highest flow rates in experiments without PRE. It is worth mentioning that the residence times without the PRE agent were longer, which positively affects polarization buildup (see Supporting Information). This further highlights the PRE agent's ability to enhance polarization buildup in this setup.

#### 3.2.3 Quantification

**FIGURE 5:**
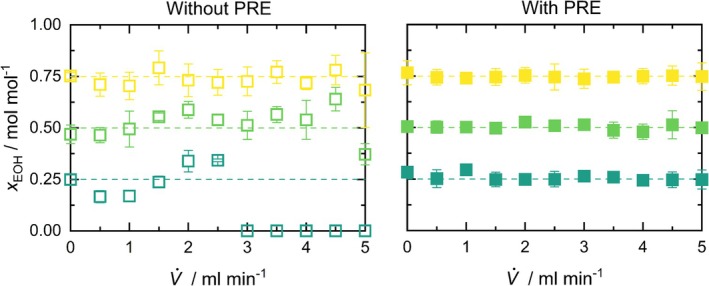
Quantification results of ethanol (EOH) for the binary Mixtures 1–3 (see Table 1) based on Equation (2) and the data shown in Figure 4. Results obtained without PRE (left) and with PRE (right) are shown as a function of flow rate. The dashed lines indicate the gravimetrically determined composition.

Figure 5 shows the results of the quantification of the ACN + EOH binary mixtures (Mixtures 1–3) based on Equation (2) and the data presented in Figure 4. The dashed line indicates the ground truth obtained from the gravimetric preparation of the samples. The influence of the flow rate on the determined composition is for Mixtures 2 and 3 less pronounced than for the signal intensity (relative polarization), since $T_{1}$ is similar for both components and leads therefore to similar decreases in signal intensity (see Figure 4). Nevertheless, the quantitative results obtained for EOH without PRE (left) are poor. A more detailed analysis of these errors is presented in the Supporting Information. In contrast, the results obtained with PRE (right) show excellent agreement with the ground truth and are independent of the flow rate up to the highest flow rates investigated. In the experiments with Mixture 1 that were carried out without PRE at the highest flow rates, no EOH signal was found (see Figure 3), leading to gross quantification errors.

### 3.3 System 2: Acetonitrile + 1,4‐Dioxane

**FIGURE 6:**
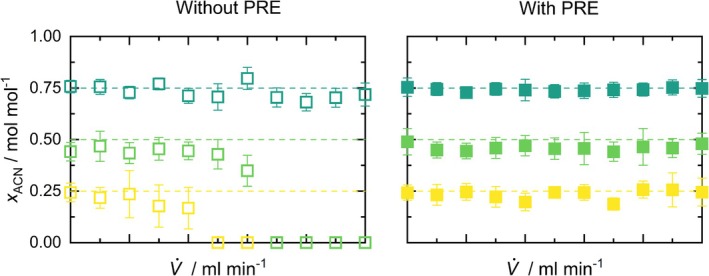
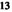
C NMR quantification results of acetonitrile (ACN) for System 2 (Mixtures 4–6) based on Equation (2) and the data shown in Figure S7 (Supporting Information). Results obtained without PRE (left) and with PRE (right) are shown as a function of flow rate. The dashed lines indicate the gravimetrically determined composition.

The results for System 2 (ACN + DIOX) are similar to those for System 1 (ACN + EOH) and are therefore only briefly discussed here. The prepolarization efficiency of System 2 is presented in the Supporting Information. The corresponding numerical data are given as well in the Supporting Information. Figure 6 shows the results of the 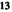
C NMR quantification of ACN in System 2 (Mixtures 4–6) based on Equation (2) and the data presented in Figure S7 (see Supporting Information). The dashed line indicates the ground truth obtained from the gravimetric preparation of the samples. As before, the combination of PENDANT and PRE yields stable composition results up to the highest flow rates investigated. Quantification by PRE in this system improves 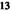
C NMR. All studied mixtures can be quantified, unlike the results without the PRE agent, which failed for Mixtures 5 and 6 at high flow rates.

### 3.4 System 3: Acetonitrile + Ethanol +1,4‐Dioxane

Quantification using 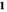
H NMR spectroscopy of the ternary mixture ACN + EOH + DIOX (Mixtures 7–10, see Table 1) is not possible due to signal overlap. Therefore, it is necessary to detec13t ^13^C nuclei. The corresponding numerical data are given in the Supporting Information.

#### 3.4.1 Prepolarization Efficiency

**FIGURE 7:**
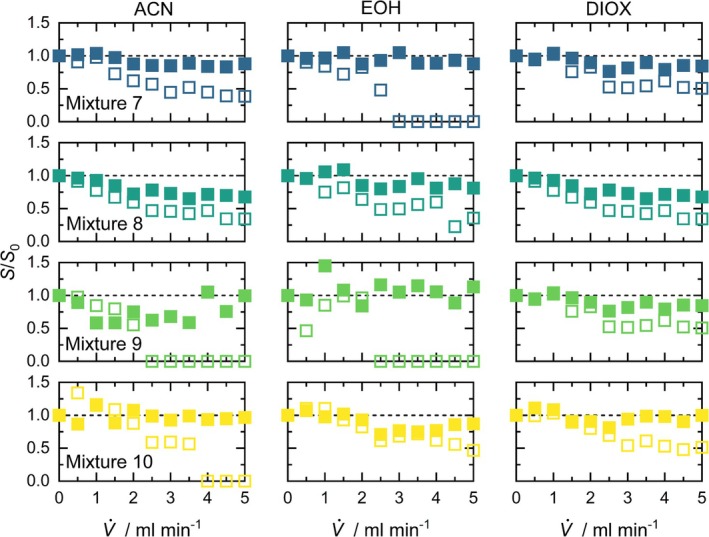
Relative polarization S/ $S_{0}$ of acetonitrile (ACN, left), ethanol (EOH, middle), and 1,4‐dioxane (DIOX, right) for the studied ACN + EOH + DIOX mixtures (Mixtures 7–10, Table 1) as a function of flow rate. Spectra were recorded using PENDANT with 16 scans. Data obtained with PRE are shown as filled symbols, data obtained without PRE as open symbols; dashed lines indicate the static reference experiments. Error bars are within symbol size.

Figure 7 shows the relative polarization S / $S_{0}$ of ACN (left), EOH (middle), and DIOX (right) for System 3 (Mixtures 7–10, see Table 1) as a function of flow rate. All spectra were recorded using PENDANT, now with 16 scans due to the lower concentrations of the components. Again, results obtained with PRE (filled symbols) are compared with measurements without PRE (open symbols) and to the static reference experiments (dashed line). With PRE, the premagnetization is in most cases enhanced to full magnetization; only at high flow rates a deviation of relative polarization is observed in some instances. In contrast, experiments performed without PRE show a pronounced decrease in relative polarization across all studied systems. In Mixture 9 and 10 ( $x_{\text{ACN}}=0.20$ mol mol 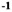), in the experiments without PRE for nearly half of the flow rates the ACN signal was so weak, that it was indistinguishable from the baseline noise. The same occurred in Mixtures 7 and 9 ( $x_{\text{EOH}}=0.20$ mol mol 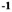) for the EOH signal. However, the signal of DIOX can be detected without PRE for all investigated flow rates and ternary mixtures. This is because of the four magnetic equivalent ${\text{CH}}_{2}$ groups of DIOX which result in one signal in the 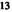
C NMR spectrum, whereas ACN and EOH have only one ${\text{CH}}_{3}$‐group each. Therefore, eight 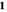
H nuclei contribute to the polarization transfer for DIOX, whereas only three 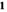
H nuclei for ACN and EOH. With PRE, the signals of ACN, EOH, and DIOX can be detected for all flow rates with enhanced polarization.

**FIGURE 8:**
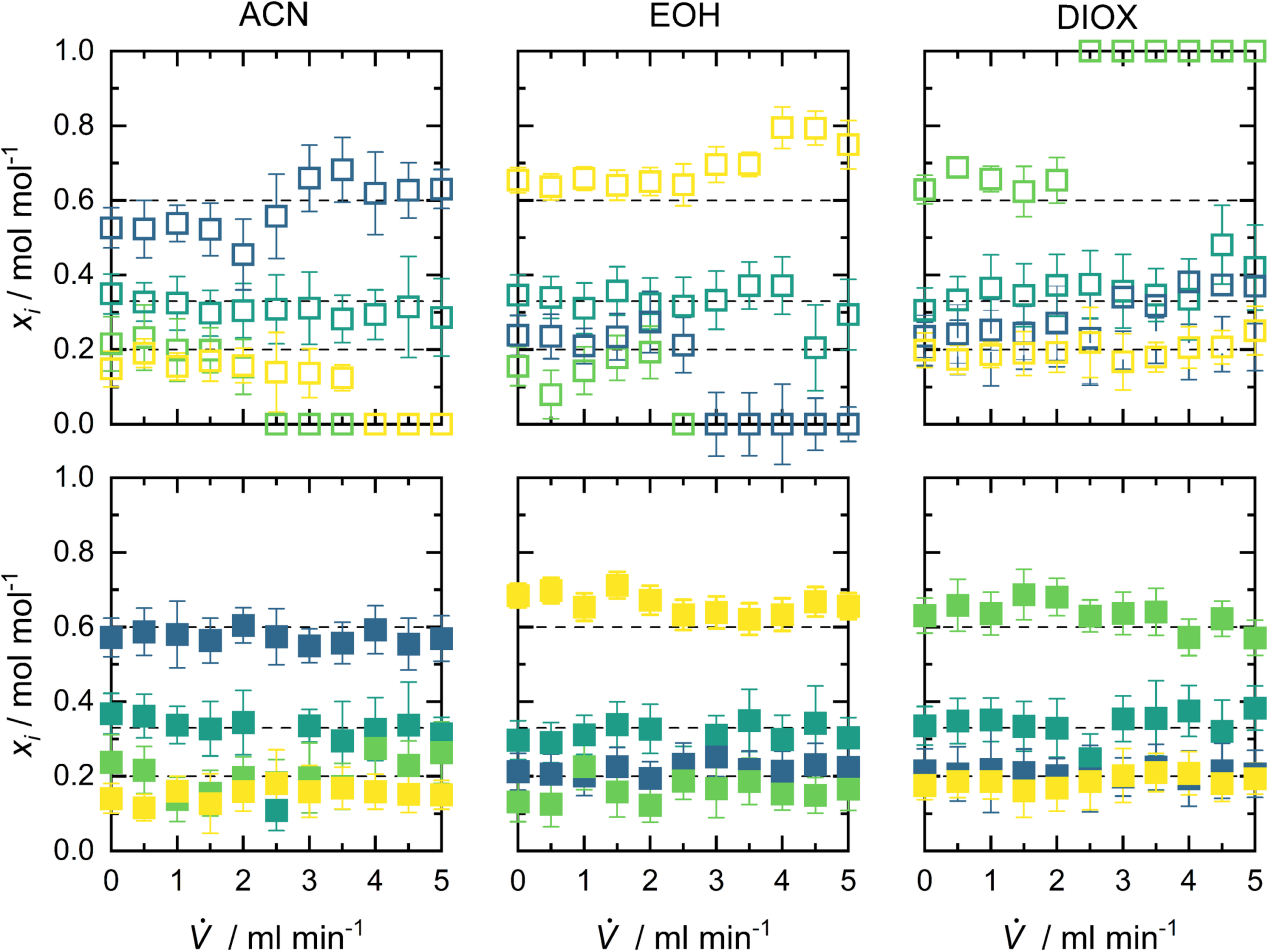
Quantification results of acetonitrile (ACN, left), ethanol (EOH, middle), and 1,4‐dioxane (DIOX, right) for the ternary Mixtures 7–10 (see Table 1) based on Equation (2) and the data shown in Figure 7. Results obtained without PRE (top) and with PRE (bottom) are shown as a function of flow rate. The dashed lines indicate the gravimetrically determined composition. The same color refers to the same mixture, see Figure 7.

Figure 8 shows the results of the quantification of ACN + EOH + DIOX in System 3 (Mixtures 7–10) based on Equation (2) and the data presented in Figure 7. Results obtained without PRE (top, open symbols) are compared with measurements with PRE (bottom, filled symbols) and to the gravimetrically determined composition (dashed line).

Without PRE, quantification is only possible with large deviations (especially for high flow rates) from the ground truth and completely fails for some flow rates (compare Figure 7). For detailed analysis of these errors, see Supporting Information. Especially for Mixtures 7, 9, and 10 quantification fails due to insufficient premagnetization of EOH and ACN for high flow rates. With PRE, quantification is possible for all mixtures and flow rates with small deviations from the ground truth. The deviations can be eliminated by accumulation of more scans if more accuracy is necessary. This highlights again the capability of our approach using PENDANT + 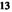
C NMR in combination with our immobilized PRE agent in continuous flow for benchtop NMR spectroscopy, where direct integration of 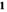
H NMR spectra fails.

## 4 Conclusions

In this work, we demonstrate that quantitative 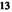
C benchtop NMR spectroscopy of fast‐flowing liquid mixtures is feasible. Two key challenges were addressed: the loss of signal intensity with increasing flow velocity due to insufficient prepolarization and the inherently low sensitivity of 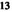
C nuclei.

Insufficient prepolarization was overcome by implementing PRE through controlled porous glass beads bearing immobilized nitroxide radicals placed upstream of the NMR detection cell. With PRE, nearly full polarization is achieved even at high flow rates in continuous‐flow benchtop 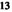
C NMR measurements. The low intrinsic sensitivity of 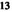
C nuclei was addressed by 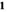
H to 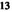
C polarization transfer using PENDANT, which substantially reduces the number of scans required for quantification, enabling rapid data acquisition.

By combining PRE and PENDANT, accurate and rapid quantification is achieved with benchtop 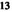
C NMR spectroscopy even for fast‐flowing samples. This substantially expands the applicability of 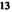
C benchtop NMR and establishes it as a viable tool for quantitative reaction monitoring and continuous process analysis.

## Author Contributions

**Sarah Mross:** conceptualization; methodology; software; data curation; investigation; validation; formal analysis; visualization; writing – original draft; writing – review and editing. **Hans Hasse:** supervision; funding acquisition; writing – review and editing. **Kerstin Münnemann:** writing – review and editing; funding acquisition; supervision; conceptualization; methodology.

## Supporting information



MRC_Supporting_Information.pdf.

Figure_S7.png.

## Data Availability

The data that support the findings of this study are available in the Supporting Information of this article.

## References

[1] M. Maiwald , H. H. Fischer , Y. K. Kim , K. Albert , and H. Hasse , “Quantitative High‐Resolution On‐Line NMR Spectroscopy in Reaction and Process Monitoring,” Journal of Magnetic Resonance 166, no. 2 (2004): 135–146, 10.1016/j.jmr.2003.09.003. 14729025

[2] A. Brächer , R. Behrens , E. von Harbou , and H. Hasse , “Application of a New Micro‐Reactor ^1^H NMR Probe Head for Quantitative Analysis of Fast Esterification Reactions,” Chemical Engineering Journal 306 (2016): 413–421, 10.1016/j.cej.2016.07.045.

[3] K. Meyer , S. Kern , N. Zientek , G. Guthausen , and M. Maiwald , “Process Control With Compact NMR,” TrAC Trends in Analytical Chemistry 83 (2016): 39–52, 10.1016/j.trac.2016.03.016.

[4] A. M. R. Hall , J. C. Chouler , A. Codina , P. T. Gierth , J. P. Lowe , and U. Hintermair , “Practical Aspects of Real‐Time Reaction Monitoring Using Multi‐Nuclear High Resolution FlowNMR Spectroscopy,” Catalysis Science & Technology 6, no. 24 (2016): 8406–8417, 10.1039/c6cy01754a.

[5] A. Friebel , E. von Harbou , K. Münnemann , and H. Hasse , “Reaction Monitoring by Benchtop NMR Spectroscopy Using a Novel Stationary Flow Reactor Setup,” Industrial & Engineering Chemistry Research 58, no. 39 (2019): 18125–18133, 10.1021/acs.iecr.9b03048.

[6] T. Rudszuck , H. Nirschl , and G. Guthausen , “Perspectives in Process Analytics Using Low Field NMR,” Journal of Magnetic Resonance 323 (2021): 106897, 10.1016/j.jmr.2020.106897. 33518174

[7] M. Kespe , E. Förster , H. Nirschl , and G. Guthausen , “Flowing Liquids in NMR: Numerical CFD Simulation and Experimental Confirmation of Magnetization Buildup,” Applied Magnetic Resonance 49, no. 7 (2018): 687–705, 10.1007/s00723-018-1016-z.

[8] G. Krüger , A. Birke , and R. Weiss , “Nuclear Magnetic Resonance (NMR) Two‐Phase Mass Flow Measurements,” Flow Measurement and Instrumentation 7, no. 1 (1996): 25–37, 10.1016/0955-5986(96)00005-2.

[9] A. Friebel , T. Specht , E. von Harbou , K. Münnemann , and H. Hasse , “Prediction of Flow Effects in Quantitative NMR Measurements,” Journal of Magnetic Resonance 312 (2020): 106683, 10.1016/j.jmr.2020.106683. 32014660

[10] A. Bara‐Estaún , M. C. Harder , C. L. Lyall , J. P. Lowe , E. Suturina , and U. Hintermair , “Paramagnetic Relaxation Agents for Enhancing Temporal Resolution and Sensitivity in Multinuclear FlowNMR Spectroscopy,” Chemistry: A European Journal 29, no. 38 (2023): e202300215, 10.1002/chem.202300215. 36946535 PMC10962566

[11] F. Dalitz , M. Maiwald , and G. Guthausen , “Considerations on the Design of Flow Cells in By‐Pass Systems for Process Analytical Applications and Its Influence on the Flow Profile Using NMR and CFD,” Chemical Engineering Science 75 (2012): 318–326, 10.1016/j.ces.2012.03.042.

[12] N. Abhyankar and V. Szalai , “Challenges and Advances in the Application of Dynamic Nuclear Polarization to Liquid‐State NMR Spectroscopy,” Journal of Physical Chemistry B 125, no. 20 (2021): 5171–5190, 10.1021/acs.jpcb.0c10937. 33960784 PMC9871957

[13] E. Ravera , C. Luchinat , and G. Parigi , “Basic Facts and Perspectives of Overhauser DNP NMR,” Journal of Magnetic Resonance 264 (2016): 78–87, 10.1016/j.jmr.2015.12.013. 26920833

[14] R. Kircher , S. Mross , H. Hasse , and K. Münnemann , “Functionalized Controlled Porous Glasses for Producing Radical‐Free Hyperpolarized Liquids by Overhauser DNP,” Molecules 27, no. 19 (2022): 6402, 10.3390/molecules27196402. 36234939 PMC9572983

[15] J. Phuong , R. Kircher , S. Mross , B. Salgado , H. Hasse , and K. Münnemann , “Quantitative Nuclear Magnetic Resonance Spectroscopy With Overhauser Dynamic Nuclear Polarization,” ChemPhysChem 26, no. 16 (2025): e202401052, 10.1002/cphc.202401052. 40682294 PMC12388179

[16] S. Jannin , J. N. Dumez , P. Giraudeau , and D. Kurzbach , “Application and Methodology of Dissolution Dynamic Nuclear Polarization in Physical, Chemical and Biological Contexts,” Journal of Magnetic Resonance 305 (2019): 41–50, 10.1016/j.jmr.2019.06.001. 31203098 PMC6616036

[17] J. Mandral , J. Phuong , J. Farjon , P. Giraudeau , K. Münnemann , and J. N. Dumez , “Ultrafast 2D Benchtop NMR Spectroscopy Enhanced by Flow Overhauser Dynamic Nuclear Polarization,” Journal of Magnetic Resonance Open 23 (2025): 100195, 10.1016/j.jmro.2025.100195.

[18] O. Semenova , P. M. Richardson , A. J. Parrott , A. Nordon , M. E. Halse , and S. B. Duckett , “Reaction Monitoring Using SABRE‐Hyperpolarized Benchtop (1 T) NMR Spectroscopy,” Analytical Chemistry 91, no. 10 (2019): 6695–6701, 10.1021/acs.analchem.9b00729. 30985110 PMC6892580

[19] P. M. Richardson , A. J. Parrott , O. Semenova , A. Nordon , S. B. Duckett , and M. E. Halse , “SABRE Hyperpolarization Enables High‐Sensitivity ^1^H and ^13^C Benchtop NMR Spectroscopy,” Analyst 143, no. 14 (2018): 3442–3450, 10.1039/c8an00596f. 29917031 PMC6040279

[20] S. Lehmkuhl , M. Wiese , L. Schubert , et al., “Continuous Hyperpolarization With Parahydrogen in a Membrane Reactor,” Journal of Magnetic Resonance 291 (2018): 8–13, 10.1016/j.jmr.2018.03.012. 29625356

[21] J. Phuong , Z. Romero , H. Hasse , and K. Münnemann , “Polarization Transfer Methods for Quantitative Analysis of Flowing Mixtures With Benchtop ^13^C NMR Spectroscopy,” Magnetic Resonance in Chemistry 62, no. 5 (2023): 398–411, 10.1002/mrc.5417. 38114253

[22] J. N. Dumez , “NMR Methods for the Analysis of Mixtures,” Chemical Communications 58, no. 100 (2022): 13855–13872, 10.1039/d2cc05053f. 36458684 PMC9753098

[23] M. Bazzoni , B. Lorandel , C. Lhoste , P. Giraudeau , and J. N. Dumez , Fast 2D NMR for Reaction and Process Monitoring (Royal Society of Chemistry, 2023), 251–283.

[24] R. Kircher , S. Mross , H. Hasse , and K. Münnemann , “Quantitative Analysis in Continuous‐Flow ^1^H Benchtop NMR Spectroscopy by Paramagnetic Relaxation Enhancement,” Applied Magnetic Resonance 54, no. 11 (2023): 1555–1569, 10.1007/s00723-023-01626-8.

[25] D. Bruck , R. Dudley , C. Fyfe , and J. Van Delden , “Sample Magnetization Using Immobilized Free Radicals for Use in Flow NMR Systems,” Journal of Magnetic Resonance (1969) 42, no. 1 (1981): 51–59, 10.1016/0022-2364(81)90009-3.

[26] Y. Zhang and D. A. Laude , “Immobilized Free‐Radical Substrates for Magnetization of Carbon‐13 Nuclei in Flow NMR Measurements,” Journal of Magnetic Resonance (1969) 87, no. 1 (1990): 46–55, 10.1016/0022-2364(90)90084-m.

[27] H. H. Fischer , M. Seiler , T. S. Ertl , et al., “Quantification Studies in Continuous‐Flow ^13^C Nuclear Magnetic Resonance Spectroscopy by Use of Immobilized Paramagnetic Relaxation Agents,” Journal of Physical Chemistry B 107, no. 20 (2003): 4879–4886, 10.1021/jp021631d.

[28] A. C. Venu , R. Nasser Din , T. Rudszuck , et al., “NMR Relaxivities of Paramagnetic Lanthanide‐Containing Polyoxometalates,” Molecules 26, no. 24 (2021): 7481, 10.3390/molecules26247481. 34946561 PMC8703889

[29] S. Cai , C. Seu , Z. Kovacs , A. D. Sherry , and Y. Chen , “Sensitivity Enhancement of Multidimensional NMR Experiments by Paramagnetic Relaxation Effects,” Journal of the American Chemical Society 128, no. 41 (2006): 13474–13478, 10.1021/ja0634526. 17031960

[30] E. Matei and A. M. Gronenborn , “ ^19^F Paramagnetic Relaxation Enhancement: A Valuable Tool for Distance Measurements in Proteins,” Angewandte Chemie International Edition 55, no. 1 (2015): 150–154, 10.1002/anie.201508464. 26510989 PMC4715678

[31] N. Schork , M. Ibrahim , A. Baksi , S. Krämer , A. K. Powell , and G. Guthausen , “NMR Relaxivities of Paramagnetic, Ultra‐High Spin Heterometallic Clusters Within Polyoxometalate Matrix as a Function of Solvent and Metal Ion,” ChemPhysChem 23, no. 19 (2022): e202200215, 10.1002/cphc.202200215. 35896954

[32] A. Swartjes , P. B. White , J. P. J. Bruekers , J. A. A. W. Elemans , and R. J. M. Nolte , “Paramagnetic Relaxation Enhancement NMR as a Tool to Probe Guest Binding and Exchange in Metallohosts,” Nature Communications 13, no. 1 (2022): 1846, 10.1038/s41467-022-29406-1. PMC8986849 35388004

[33] F. X. Theillet , A. Binolfi , S. Liokatis , S. Verzini , and P. Selenko , “Paramagnetic Relaxation Enhancement to Improve Sensitivity of Fast NMR Methods: Application to Intrinsically Disordered Proteins,” Journal of Biomolecular NMR 51, no. 4 (2011): 487–495, 10.1007/s10858-011-9577-2. 22008951

[34] G. Guthausen , J. R. Machado , B. Luy , et al., “Characterisation and Application of Ultra‐High Spin Clusters as Magnetic Resonance Relaxation Agents,” Dalton Transactions 44, no. 11 (2015): 5032–5040, 10.1039/c4dt02916j. 25670214

[35] J. Homer and M. C. Perry , “New Method for NMR Signal Enhancement by Polarization Transfer, and Attached Nucleus Testing,” Journal of the Chemical Society, Chemical Communications 4 (1994): 373, 10.1039/c39940000373.

[36] J. Homer and M. C. Perry , “Enhancement of the NMR Spectra of Insensitive Nuclei Using PENDANT With Long‐Range Coupling Constants,” Journal of the Chemical Society, Perkin Transactions 2, no. 3 (1995): 533, 10.1039/p29950000533.

[37] J. Phuong , S. Mross , D. Bellaire , H. Hasse , and K. Münnemann , “Determination of Self‐Diffusion Coefficients in Mixtures With Benchtop ^13^C NMR Spectroscopy via Polarization Transfer,” Magnetic Resonance in Chemistry 62, no. 5 (2023): 386–397, 10.1002/mrc.5412. 38014888

[38] J. Phuong , B. Salgado , T. Labusch , H. Hasse , and K. Münnemann , “Overhauser Dynamic Nuclear Polarization Enables Single Scan Benchtop ^13^C NMR Spectroscopy in Continuous‐Flow,” Analytical Chemistry 97, no. 8 (2025): 4308–4317, 10.1021/acs.analchem.4c03985. 39984167 PMC11883742

