# Supplementary material for: Enabling Quantitative Benchtop 13C NMR Spectroscopy in Fast Continuous Flow
Source: Magn Reson Chem. 2026 May 15;64(9):758–67. doi: 10.1002/mrc.70119 (PMC13432203; doi:10.1002/mrc.70119)
Supplement: Supplementary file 1 — MRC_Supporting_Information.pdf. [file MRC-64-758-s001.pdf]

Supporting Information: Enabling Quantitative Benchtop ^{13}C NMR Spectroscopy in Fast Continuous-Flow

Sarah Mross,[†,‡] Hans Hasse,[†,‡] and Kerstin Münnemann[*,†,‡]

†*Laboratory of Engineering Thermodynamics (LTD), RPTU, 67663 Kaiserslautern, Germany*

‡*Laboratory of Advanced Spin Engineering - Magnetic Resonance (LASE-MR), RPTU, 67663 Kaiserslautern, Germany*

E-mail: *kerstin.muennemann@rptu.de

1 Experimental Section

Flow Velocities and Residence Times in the Setup

Table S1 gives a detailed overview of the flow velocity ν and residence time τ in the pre-magnetization zone of our setup for the empty PRE agent cell and the filled PRE agent cell. To calculate the volume of the filled cell, ideal dense packing of the fixed bed is assumed with a void fraction of 0.26. Furthermore, the mean flow velocity $\bar{\nu}$, calculated by Eq. (S1), and the total residence time τ_{tot} refers to the combined contribution of the PRE agent cell and the connected capillary.

$$\bar{\nu} = \frac{\nu_1 l_1 + \nu_2 l_2}{l_1 + l_2} \tag{S1}$$

Here, ν_1 and ν_2 are the flow velocities in the capillary (1) and the PRE agent cell (2) with the lengths l_1 and l_2 of 83 mm and 17 mm, respectively (see main part). The flow velocities in the filled PRE agent cell are higher, which results in shorter residence times, respectively.

Table S1: Overview of the flow velocity ν, mean flow velocity $\bar{\nu}$, residence time τ and total residence time τ_{tot} in the pre-magnetization zone of the experimental setup for the capillary and both the empty and the filled PRE agent cell.

Flow property	Flow rate / mL min^{-1}	Capillary	PRE agent cell (empty)	PRE agent cell (filled)
ν / m s^{-1}	0.5	0.011	0.003	0.010
$\bar{\nu}$ / m s^{-1}		-	0.009	0.011
τ / s		7.823	6.409	1.666
τ_{tot} / s		-	14.23	9.489
ν / m s^{-1}	1.0	0.021	0.005	0.020
$\bar{\nu}$ / m s^{-1}		-	0.019	0.021
τ / s		3.911	3.204	0.833
τ_{tot} / s		-	7.116	4.744
ν / m s^{-1}	1.5	0.032	0.008	0.031
$\bar{\nu}$ / m s^{-1}		-	0.028	0.032
τ / s		2.608	2.136	0.555
τ_{tot} / s		-	4.744	3.163
ν / m s^{-1}	2.0	0.042	0.011	0.041
$\bar{\nu}$ / m s^{-1}		-	0.037	0.042
τ / s		1.956	1.602	0.417
τ_{tot} / s		-	3.558	2.372
ν / m s^{-1}	2.5	0.053	0.013	0.051
$\bar{\nu}$ / m s^{-1}		-	0.046	0.053
τ / s		1.565	1.282	0.333
τ_{tot} / s		-	2.846	1.898
ν / m s^{-1}	3.0	0.064	0.016	0.061
$\bar{\nu}$ / m s^{-1}		-	0.056	0.063
τ / s		1.304	1.068	0.278
τ_{tot} / s		-	2.372	1.581
ν / m s^{-1}	3.5	0.074	0.019	0.071
$\bar{\nu}$ / m s^{-1}		-	0.065	0.074
τ / s		1.118	0.916	0.238
τ_{tot} / s		-	2.033	1.356
ν / m s^{-1}	4.0	0.085	0.021	0.082
$\bar{\nu}$ / m s^{-1}		-	0.074	0.084
τ / s		0.978	0.801	0.208
τ_{tot} / s		-	1.779	1.186
ν / m s^{-1}	4.5	0.095	0.024	0.092
$\bar{\nu}$ / m s^{-1}		-	0.083	0.095
τ / s		0.869	0.712	0.185
τ_{tot} / s		-	1.581	1.054
ν / m s^{-1}	5.0	0.106	0.027	0.102
$\bar{\nu}$ / m s^{-1}		-	0.093	0.105
τ / s		0.782	0.641	0.167
τ_{tot} / s		-	1.423	0.949

Error Calculation

The relative deviation δ_{rel} of the mole fraction x_i of component i was calculated according to Eq. (S2), where the x_i^{true} is the ground truth from gravimetrical preparation (see Table 2 in the main part) and x_i^{exp} is the mole fraction x_i obtained from NMR measurements.

$$\delta_{\mathrm{rel},i} = \frac{x_i^{\mathrm{exp}} - x_i^{\mathrm{true}}}{x_i^{\mathrm{true}}} * 100\% \tag{S2}$$

2 ^{1}H Relaxation Times T_1

The T_1 measurements were performed with a 42.5 MHz Benchtop from Magritek using the inversion recovery experiment. The fit of the T_1 times was done with MATLAB. The results are the arithmetic values of three repeated measurements. For more details on the measurement see Ref.[1] Table S2 gives the ^{1}H spin-lattice relaxation times T_1^0 of system 1-3 without the PRE agent. Only ^{1}H T_1 times were measured, as the relaxation delay in the PENDANT + ^{13}C NMR experiments depends only on the ^{1}H T_1 time due to the polarization transfer from ^{1}H to ^{13}C nuclei.

Table S2: ^{1}H relaxation times (T_1^0) of system 1-3 without the PRE agent at 42.5 MHz.

System	Mixture	T_1^0 / s		
		ACN (CH_3)	EOH (CH_3)	DIOX (CH_2)
1	1	3.14 (± 0.03)	2.82 (± 0.01)	-
	2	3.52 (± 0.01)	3.17 (± 0.01)	-
	3	3.87 (± 0.02)	3.66 (± 0.05)	-
2	4	3.14 (± 0.01)	-	2.97 (± 0.02)
	5	3.37 (± 0.01)	-	3.17 (± 0.01)
	6	3.70 (± 0.01)	-	3.48 (± 0.01)
3	7	3.05 (± 0.01)	2.82 (± 0.01)	3.05 (± 0.01)
	8	2.90 (± 0.01)	2.89 (± 0.01)	2.90 (± 0.01)
	9	3.33 (± 0.01)	3.11 (± 0.02)	3.33 (± 0.01)
	10	3.63 (± 0.03)	3.36 (± 0.03)	3.42 (± 0.02)

3 System 1: Acetonitrile + Ethanol

^{1}H NMR of System 1

Figure S1 shows the relative polarization S/S_0 of ^{1}H of ACN (left) and EOH (right) for the pure solvents and system 1 (Mixtures 1-3; see Table 2 main part) as a function of flow rate. All spectra were recorded using ^{1}H NMR with 1 scan. Results obtained with PRE (filled symbols) are compared to measurements without PRE (open symbols) and to the static reference experiments (dashed line). Here, the same trends are present for the PENDANT + ^{13}C NMR results. The polarization of ACN and EOH is enhanced with PRE and is almost constant for all investigated flow rates and mixtures.

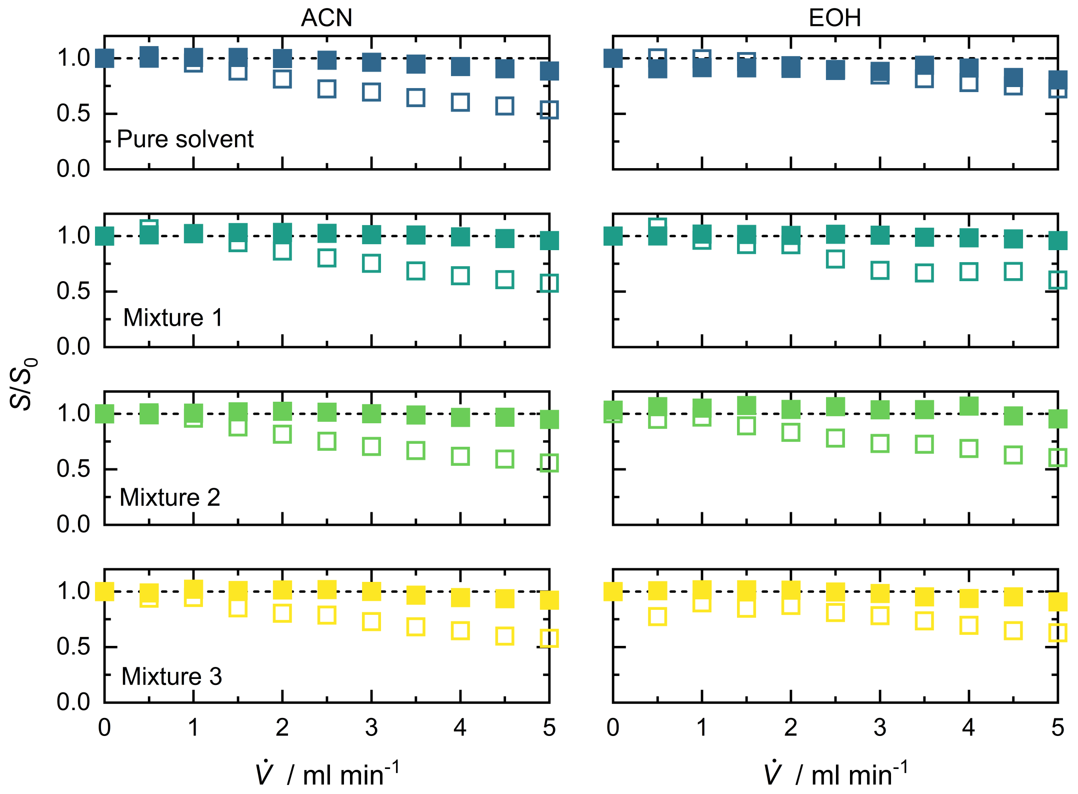

Figure S1: ^{1}H NMR relative polarization S/S_0 of acetonitrile (ACN, left) and ethanol (EOH, right) for the pure solvents and system 1 (Mixtures 1-3, see Table 2 main part) as a function of flow rate. Spectra were recorded using ^{1}H NMR with 1 scan. Data obtained with PRE are shown as filled symbols, data obtained without PRE as open symbols; dashed lines indicate the static reference experiments. Error bars are within symbol size.

Figure S2 shows the results of the ^{1}H NMR quantification of EOH in system 1 (Mixtures 1-3) based on Eq. (2) (see main part) and the data presented in Figure S1. The dashed line indicates the ground truth obtained from the gravimetric preparation of the samples. The improvement in quantification by PRE in this system for ^{1}H NMR is only small due to the similar ^{1}H T_1 times of both components in Mixtures 1-3 which leads to almost the same decrease of the recorded ^{1}H NMR signals.

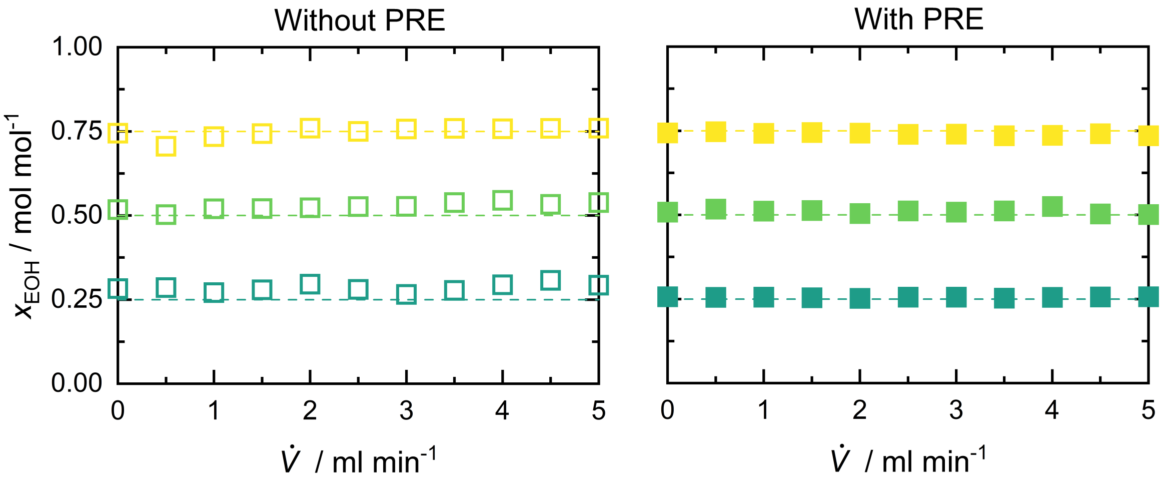

Figure S2: ^{1}H NMR quantification results of ethanol (EOH) for system 1 (Mixtures 1-3) based on Eq. (2) of the data shown in Figure S1. Results obtained without PRE (left) and with PRE (right) are shown as a function of flow rate. The dashed lines indicate the gravimetrically determined composition. Error bars are within symbol size.

Figure S3 and S4 show the relative deviation δ_{rel} of the ^{1}H NMR results of EOH and ACN in system 1 (Mixtures 1-3) as a function of flow rate. With the PRE agent, the relative deviations of the obtained mole fractions of EOH and ACN for Mixtures 1-3 are significantly reduced.

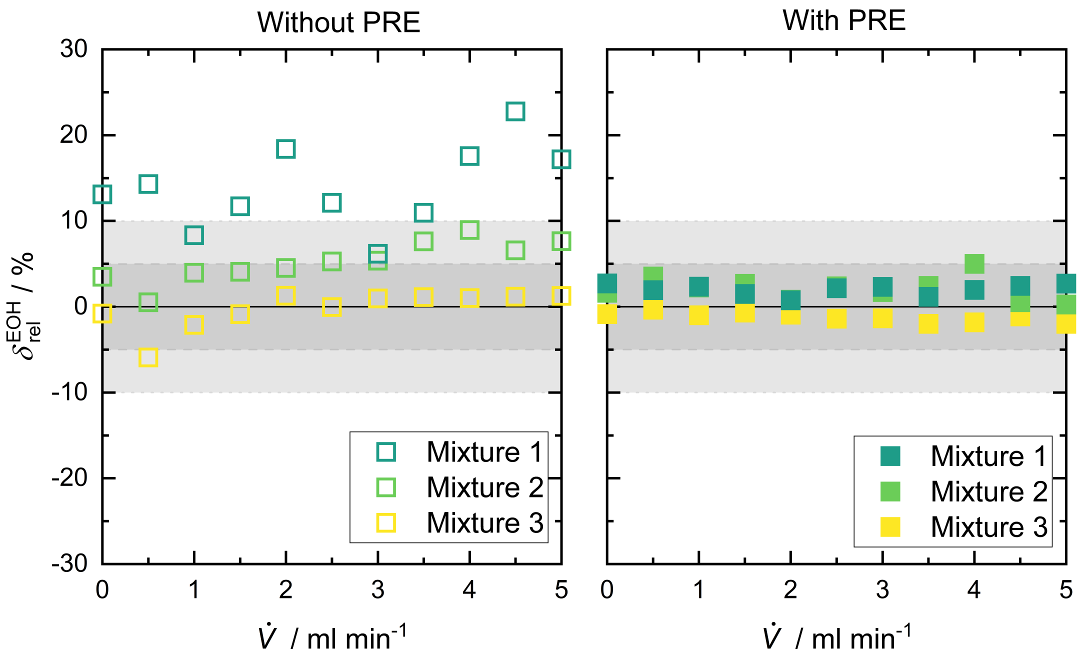

Figure S3: Relative deviations δ_{rel} of the quantification based on ^{1}H NMR results of ethanol (EOH) in system 1 (Mixtures 1-3) calculated according to Eq. (S2) of the data shown in Figure S2. δ_{rel} calculated for results without PRE (left) and with PRE (right) are shown as a function of flow rate. The shaded regions represent relative deviations of 5% (dark gray) and 10% (light gray) from the gravimetrically determined composition.

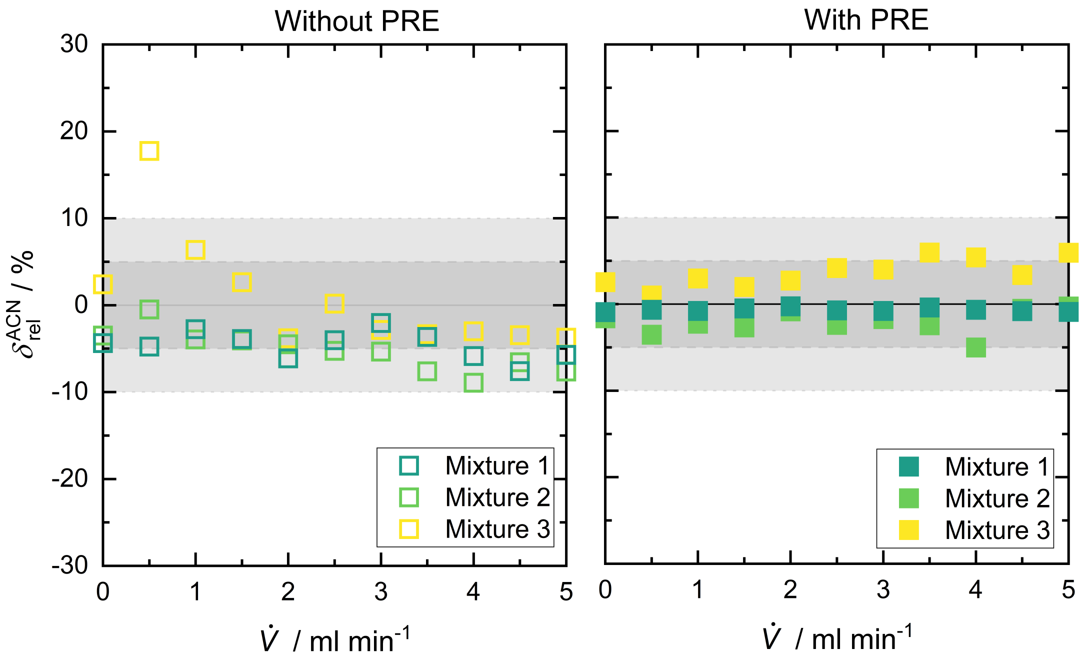

Figure S4: Relative deviations δ_{rel} of the quantification based on ^{1}H NMR results of acetonitrile (ACN) in system 1 (Mixtures 1-3) calculated according to Eq. (S2) of the data shown in Figure S2. δ_{rel} calculated for results without PRE (left) and with PRE (right) are shown as a function of flow rate. The shaded regions represent relative deviations of 5% (dark gray) and 10% (light gray) from the gravimetrically determined composition.

Tables S3 and S4 give the numerical data of the normalized integrals obtained by ^{1}H NMR of pure ACN and in system 1 (Mixtures 1-3) without and with the PRE agent for the investigated flow rates.

Table S3: Numerical data of the normalized integrals for ^{1}H NMR of pure acetonitrile (ACN) and in system 1 (Mixtures 1-3) without the PRE agent at 42.5 MHz.

Flow rate / mL min^{-1}	$A_{\texttt{ACN}}$ without PRE			
	pure	mixture 1	mixture 2	mixture 3
0.0	24.38 (± 0.038)	16.15 (± 0.050)	11.62 (± 0.009)	6.243 (± 0.011)
0.5	25.04 (± 0.007)	17.19 (± 0.010)	11.71 (± 0.011)	5.864 (± 0.082)
1.0	23.40 (± 0.017)	16.48 (± 0.010)	11.15 (± 0.012)	5.905 (± 0.005)
1.5	21.62 (± 0.013)	15.17 (± 0.070)	10.24 (± 0.008)	5.318 (± 0.006)
2.0	19.86 (± 0.013)	13.95 (± 0.010)	9.476 (± 0.006)	5.018 (± 0.004)
2.5	17.72 (± 0.048)	12.95 (± 0.030)	8.742 (± 0.013)	4.918 (± 0.011)
3.0	16.12 (± 0.154)	12.18 (± 0.010)	8.186 (± 0.006)	4.549 (± 0.006)
3.5	15.57 (± 0.016)	11.06 (± 0.010)	7.757 (± 0.013)	4.255 (± 0.008)
4.0	14.62 (± 0.022)	10.37 (± 0.010)	7.160 (± 0.010)	4.040 (± 0.006)
4.5	13.93 (± 0.028)	9.789 (± 0.020)	6.864 (± 0.010)	3.733 (± 0.006)
5.0	13.07 (± 0.022)	9.277 (± 0.020)	6.460 (± 0.010)	3.610 (± 0.008)

Table S4: Numerical data of the normalized integrals for ^{1}H NMR of pure acetonitrile (ACN) and in system 1 (Mixtures 1-3) with the PRE agent at 42.5 MHz.

Flow rate / mL min^{-1}	$A_{\texttt{ACN}}$ with PRE			
	pure	mixture 1	mixture 2	mixture 3
0.0	25.21 (± 0.080)	18.08 (± 0.008)	12.33 (± 0.016)	6.538 (± 0.010)
0.5	25.40 (± 0.072)	18.29 (± 0.070)	12.23 (± 0.015)	6.485 (± 0.038)
1.0	27.87 (± 0.013)	18.49 (± 0.040)	12.40 (± 0.045)	6.435 (± 0.009)
1.5	27.06 (± 0.009)	18.68 (± 0.015)	12.56 (± 0.012)	6.430 (± 0.042)
2.0	26.92 (± 0.033)	18.68 (± 0.026)	12.62 (± 0.057)	6.428 (± 0.010)
2.5	27.98 (± 0.013)	18.43 (± 0.013)	12.51 (± 0.011)	6.438 (± 0.022)
3.0	27.20 (± 0.014)	18.45 (± 0.006)	12.35 (± 0.039)	6.575 (± 0.011)
3.5	26.43 (± 0.015)	18.18 (± 0.091)	12.20 (± 0.015)	6.331 (± 0.028)
4.0	26.43 (± 0.009)	17.88 (± 0.013)	11.94 (± 0.027)	6.182 (± 0.009)
4.5	25.30 (± 0.008)	17.57 (± 0.010)	11.96 (± 0.016)	6.121 (± 0.025)
5.0	25.10 (± 0.007)	17.39 (± 0.013)	11.71 (± 0.021)	6.027 (± 0.005)

Tables S5 and S6 give the numerical data of the normalized integrals obtained by ^{1}H NMR of pure EOH and in system 1 (Mixtures 1-3) without and with the PRE agent for the investigated flow rates.

Table S5: Numerical data of the normalized integrals for ^{1}H NMR of pure ethanol (EOH) and in system 1 (Mixtures 1-3) without the PRE agent at 42.5 MHz.

Flow rate / mL min^{-1}	$A_{\mathtt{EOH}}$ without PRE			
	pure	mixture 1	mixture 2	mixture 3
0.0	18.28 (± 0.018)	6.366 (± 0.050)	12.46 (± 0.032)	18.15 (± 0.055)
0.5	18.33 (± 0.048)	6.875 (± 0.020)	11.83 (± 0.046)	14.06 (± 0.212)
1.0	18.18 (± 0.022)	6.120 (± 0.010)	12.07 (± 0.022)	16.30 (± 0.034)
1.5	17.71 (± 0.022)	5.877 (± 0.030)	11.11 (± 0.015)	15.41 (± 0.036)
2.0	17.05 (± 0.033)	5.865 (± 0.030)	10.37 (± 0.015)	15.85 (± 0.020)
2.5	16.25 (± 0.017)	5.043 (± 0.030)	9.714 (± 0.030)	14.72 (± 0.040)
3.0	15.51 (± 0.029)	4.399 (± 0.050)	9.113 (± 0.024)	14.18 (± 0.030)
3.5	14.91 (± 0.042)	4.246 (± 0.040)	9.037 (± 0.026)	13.36 (± 0.033)
4.0	14.28 (± 0.024)	4.316 (± 0.020)	8.565 (± 0.040)	12.62 (± 0.030)
4.5	13.72 (± 0.033)	4.335 (± 0.020)	7.830 (± 0.029)	11.74 (± 0.017)
5.0	13.23 (± 0.032)	3.844 (± 0.030)	7.528 (± 0.023)	11.39 (± 0.029)

Table S6: Numerical data of the normalized integrals for ^{1}H NMR of pure ethanol (EOH) and in system 1 (Mixtures 1-3) with the PRE agent at 42.5 MHz.

Flow rate / mL min^{-1}	$A_{\mathtt{EOH}}$ with PRE			
	pure	mixture 1	mixture 2	mixture 3
0.0	25.23 (± 0.105)	6.890 (± 0.034)	12.74 (± 0.043)	18.44 (± 0.056)
0.5	22.85 (± 0.046)	7.204 (± 0.094)	13.13 (± 0.112)	18.35 (± 0.084)
1.0	23.10 (± 0.040)	7.138 (± 0.034)	12.97 (± 0.105)	18.78 (± 0.053)
1.5	23.08 (± 0.069)	7.052 (± 0.033)	13.25 (± 0.048)	19.25 (± 0.037)
2.0	22.95 (± 0.046)	6.945 (± 0.022)	12.83 (± 0.058)	18.50 (± 0.038)
2.5	22.67 (± 0.055)	7.397 (± 0.036)	13.13 (± 0.032)	18.60 (± 0.106)
3.0	22.25 (± 0.022)	7.171 (± 0.036)	12.78 (± 0.086)	17.92 (± 0.040)
3.5	23.65 (± 0.043)	7.336 (± 0.120)	12.81 (± 0.070)	17.567 (± 0.030)
4.0	23.04 (± 0.036)	6.977 (± 0.044)	13.19 (± 0.049)	17.28 (± 0.029)
4.5	20.86 (± 0.063)	7.065 (± 0.020)	12.09 (± 0.058)	17.57 (± 0.105)
5.0	20.32 (± 0.060)	7.407 (± 0.049)	11.77 (± 0.048)	16.73 (± 0.023)

^{13}C NMR of System 1

Figures S5 and S6 show the relative deviation δ_{rel} of the ^{13}C NMR results, recorded with PENDANT, for EOH and ACN quantification in system 1 (Mixtures 1-3) as a function of flow rate. With the PRE agent, the relative deviations of the obtained mole fractions of EOH and ACN for Mixtures 1-3 are significantly reduced.

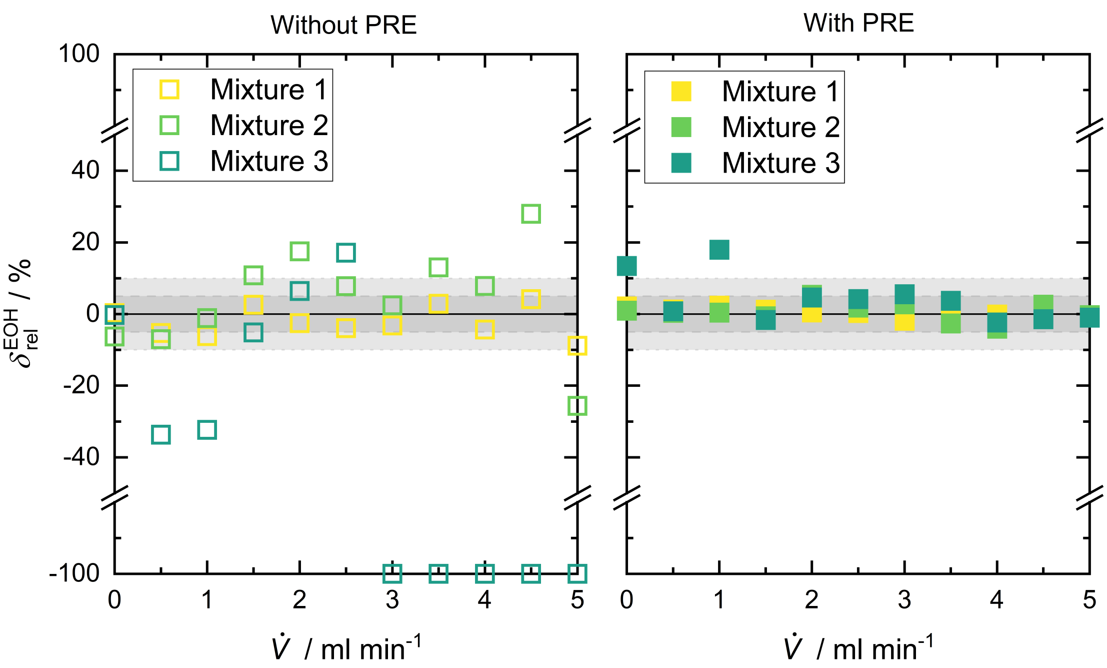

Figure S5: Relative deviations δ_{rel} of the quantification based on ^{13}C NMR results, recorded with PENDANT, of ethanol (EOH) in system 1 (Mixtures 1-3) calculated according to Eq. (S2) of the data shown in Figure 5. δ_{rel} calculated for results without PRE (left) and with PRE (right) are shown as a function of flow rate. The shaded regions represent relative deviations of 5% (dark gray) and 10% (light gray) from the gravimetrically determined composition.

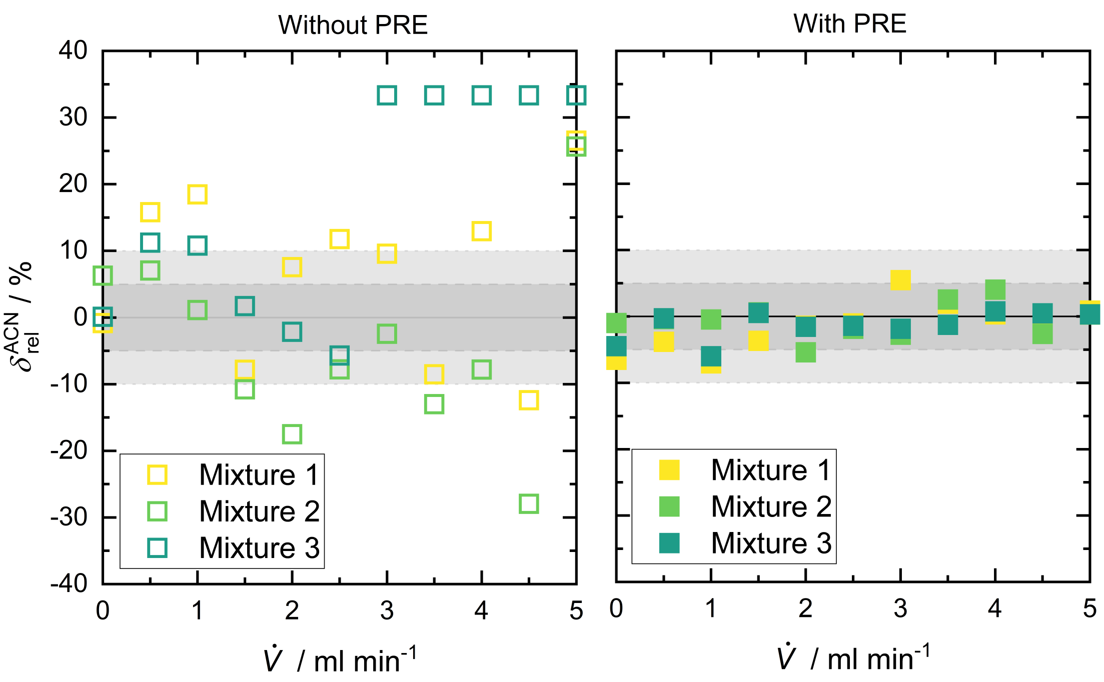

Figure S6: Relative deviations δ_{rel} of the quantification based on ^{13}C NMR results, recorded with PENDANT, of acetonitrile (ACN) in system 1 (Mixtures 1-3) calculated according to Eq. (S2) of the data shown in Figure 5. δ_{rel} calculated for results without PRE (left) and with PRE (right) are shown as a function of flow rate. The shaded regions represent relative deviations of 5% (dark gray) and 10% (light gray) from the gravimetrically determined composition.

Tables S7 and S8 give the numerical data of the normalized integrals obtained by ^{13}C NMR, recorded with PENDANT, of pure ACN and in system 1 (Mixtures 1-3) without and with the PRE agent for the investigated flow rates.

Table S7: Numerical data of the normalized integrals for ^{13}C NMR, recorded with PENDANT, of pure acetonitrile (ACN) and in system 1 (Mixtures 1-3) without the PRE agent at 42.5 MHz.

Flow rate / mL min^{-1}	$A_{ACN} \times 10^{-3}$ without PRE pure	mixture 1	mixture 2	mixture 3
0.0	5.761 (± 0.321)	4.278 (± 0.284)	3.076 (± 0.479)	1.538 (± 0.094)
0.5	5.505 (± 0.537)	4.538 (± 0.261)	3.144 (± 0.269)	1.534 (± 0.334)
1.0	5.455 (± 0.598)	3.825 (± 0.192)	2.600 (± 0.820)	1.477 (± 0.416)
1.5	4.825 (± 1.082)	3.410 (± 0.455)	1.877 (± 0.018)	1.142 (± 0.363)
2.0	4.583 (± 0.775)	1.918 (± 0.262)	1.282 (± 0.174)	1.199 (± 0.468)
2.5	4.035 (± 0.877)	2.108 (± 0.027)	1.174 (± 0.089)	1.227 (± 0.359)
3.0	4.308 (± 1.090)	1.680 (± 0.056)	1.215 (± 0.141)	1.005 (± 0.345)
3.5	3.203 (± 0.561)	1.554 (± 0.495)	0.917 (± 0.018)	0.827 (± 0.235)
4.0	3.255 (± 0.586)	1.624 (± 0.443)	0.930 (± 0.302)	0.912 (± 0.129)
4.5	3.442 (± 0.627)	2.239 (± 0.057)	0.817 (± 0.134)	0.724 (± 0.287)
5.0	2.409 (± 0.703)	1.439 (± 0.071)	1.035 (± 0.202)	0.714 (± 0.600)

Table S8: Numerical data of the normalized integrals for ^{13}C NMR, recorded with PENDANT, of pure acetonitrile (ACN) and in system 1 (Mixtures 1-3) with the PRE agent at 42.5 MHz.

Flow rate / mL min^{-1}	$A_{ACN} \times 10^{-3}$ with PRE pure	mixture 1	mixture 2	mixture 3
0.0	6.627 (± 0.740)	4.392 (± 0.140)	2.823 (± 0.248)	1.322 (± 0.428)
0.5	6.504 (± 0.502)	4.571 (± 0.329)	2.887 (± 0.203)	1.390 (± 0.236)
1.0	6.858 (± 0.782)	4.074 (± 0.436)	2.764 (± 0.254)	1.169 (± 0.131)
1.5	6.607 (± 0.991)	3.692 (± 0.374)	2.887 (± 0.193)	1.313 (± 0.262)
2.0	6.317 (± 0.766)	3.437 (± 0.477)	2.611 (± 0.187)	1.256 (± 0.256)
2.5	6.816 (± 0.967)	3.701 (± 0.311)	2.862 (± 0.117)	1.326 (± 0.288)
3.0	6.694 (± 0.847)	4.482 (± 0.200)	2.440 (± 0.148)	1.320 (± 0.312)
3.5	6.001 (± 0.684)	4.030 (± 0.200)	2.490 (± 0.109)	1.189 (± 0.126)
4.0	6.052 (± 1.510)	4.439 (± 0.531)	2.683 (± 0.356)	1.127 (± 0.211)
4.5	6.677 (± 0.854)	4.541 (± 0.091)	2.084 (± 0.157)	1.062 (± 0.263)
5.0	6.328 (± 0.981)	4.295 (± 0.284)	2.518 (± 0.124)	0.982 (± 0.347)

Tables S9 and S10 give the numerical data of the normalized integrals obtained by ^{13}C NMR, recorded with PENDANT, of pure EOH and in system 1 (Mixtures 1-3) without and with the PRE agent for the investigated flow rates.

Table S9: Numerical data of the normalized integrals for ^{13}C NMR, recorded with PENDANT, of pure ethanol (EOH) and in system 1 (Mixtures 1-3) without the PRE agent at 42.5 MHz.

Flow rate / mL min^{-1}	$A_{\texttt{EOH}} \times 10^{-3}$ without PRE			
	pure	mixture 1	mixture 2	mixture 3
0.0	5.913 (± 0.433)	1.420 (± 0.144)	2.714 (± 0.256)	4.666 (± 0.281)
0.5	4.626 (± 0.288)	0.902 (± 0.185)	2.730 (± 0.328)	3.764 (± 0.665)
1.0	3.961 (± 0.510)	0.778 (± 0.135)	2.543 (± 0.376)	3.511 (± 0.546)
1.5	3.794 (± 0.187)	1.060 (± 0.093)	2.330 (± 0.164)	3.816 (± 1.478)
2.0	4.272 (± 0.327)	0.695 (± 0.295)	1.827 (± 0.181)	3.259 (± 0.314)
2.5	3.849 (± 0.420)	0.873 (± 0.321)	1.372 (± 0.077)	3.165 (± 0.320)
3.0	3.746 (± 0.780)	0	1.276 (± 0.317)	2.664 (± 0.207)
3.5	3.890 (± 0.438)	0	1.192 (± 0.186)	2.790 (± 0.372)
4.0	3.647 (± 0.613)	0	1.088 (± 0.218)	2.318 (± 0.180)
4.5	3.457 (± 0.638)	0	1.451 (± 0.272)	2.583 (± 0.335)
5.0	2.825 (± 0.441)	0	0.613 (± 0.065)	1.544 (± 0.023)

Table S10: Numerical data of the normalized integrals for ^{13}C NMR, recorded with PENDANT, of pure ethanol (EOH) and in system 1 (Mixtures 1-3) with the PRE agent at 42.5 MHz.

Flow rate / mL min^{-1}	$A_{\texttt{EOH}} \times 10^{-3}$ with PRE			
	pure	mixture 1	mixture 2	mixture 3
0.0	5.330 (± 1.032)	1.739 (± 0.108)	2.879 (± 0.071)	4.335 (± 0.121)
0.5	5.278 (± 0.982)	1.540 (± 0.337)	2.909 (± 0.282)	4.391 (± 0.536)
1.0	5.311 (± 1.531)	1.705 (± 0.078)	2.789 (± 0.123)	3.863 (± 0.058)
1.5	5.148 (± 1.406)	1.205 (± 0.171)	2.852 (± 0.257)	4.139 (± 0.093)
2.0	5.205 (± 1.285)	1.217 (± 0.234)	2.909 (± 0.032)	3.839 (± 0.086)
2.5	4.859 (± 1.247)	1.304 (± 0.115)	2.970 (± 0.056)	4.034 (± 0.503)
3.0	5.171 (± 1.854)	1.607 (± 0.127)	2.577 (± 0.154)	3.687 (± 0.057)
3.5	5.445 (± 1.216)	1.411 (± 0.127)	2.365 (± 0.354)	3.475 (± 0.214)
4.0	5.199 (± 0.967)	1.434 (± 0.241)	2.474 (± 0.165)	3.366 (± 0.214)
4.5	5.463 (± 2.021)	1.486 (± 0.369)	2.197 (± 0.577)	3.220 (± 0.109)
5.0	5.252 (± 1.109)	1.413 (± 0.179)	2.503 (± 0.078)	2.912 (± 0.008)

4 System 2: Acetonitrile + 1,4-Dioxane

^{1}H NMR in System 2

Figure S7 shows the relative polarization S/S_0 of ^{1}H of ACN (left) and DIOX (right) for the pure solvents and system 2 (Mixtures 4-6; see Table 2 main part) as a function of flow rate. All spectra were recorded using ^{1}H NMR with 1 scan. Results obtained with PRE (filled symbols) are compared to measurements without PRE (open symbols) and to the static reference experiments (dashed line). Here, the same trends are present for the PENDANT + ^{13}C NMR results. The polarization of ACN and DIOX is enhanced with PRE and is almost constant for all investigated flow rates and mixtures.

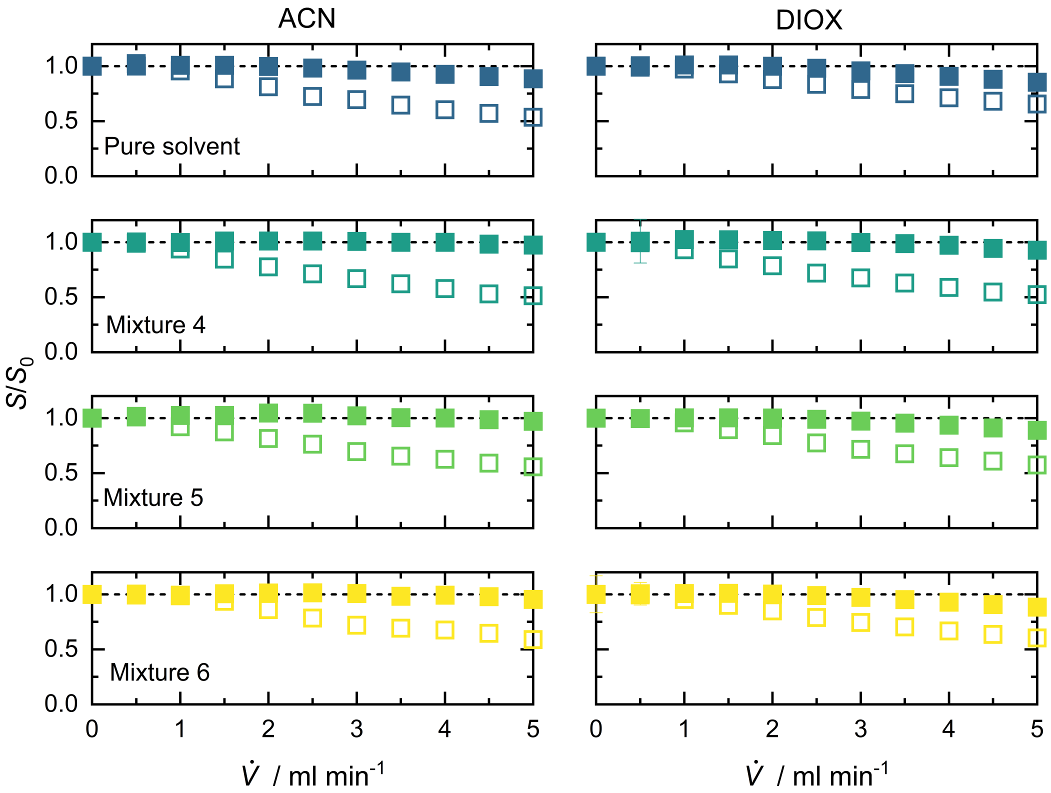

Figure S7: ^{1}H NMR relative polarization S/S_0 of acetonitrile (ACN, left) and 1,4-dioxane (DIOX, right) for the pure solvents and system 2 (Mixtures 4-6) as a function of flow rate. Spectra were recorded using ^{1}H NMR with 1 scan. Data obtained with PRE are shown as filled symbols, data obtained without PRE as open symbols; dashed lines indicate the static reference experiments. Error bars are within symbol size.

Figure S8 shows the results of the ^{1}H NMR quantification of ACN in system 2 (Mixtures 4-6) based on Eq. (2) (see main part) and the data presented in Figure S7. The dashed line indicates the ground truth from the gravimetric preparation of the samples. Again, the improvement in quantification by PRE in this system for ^{1}H NMR is only small due to the similar ^{1}H T_1 times of both components in Mixtures 4-6 which leads to almost the same decrease of the recorded ^{1}H NMR signals.

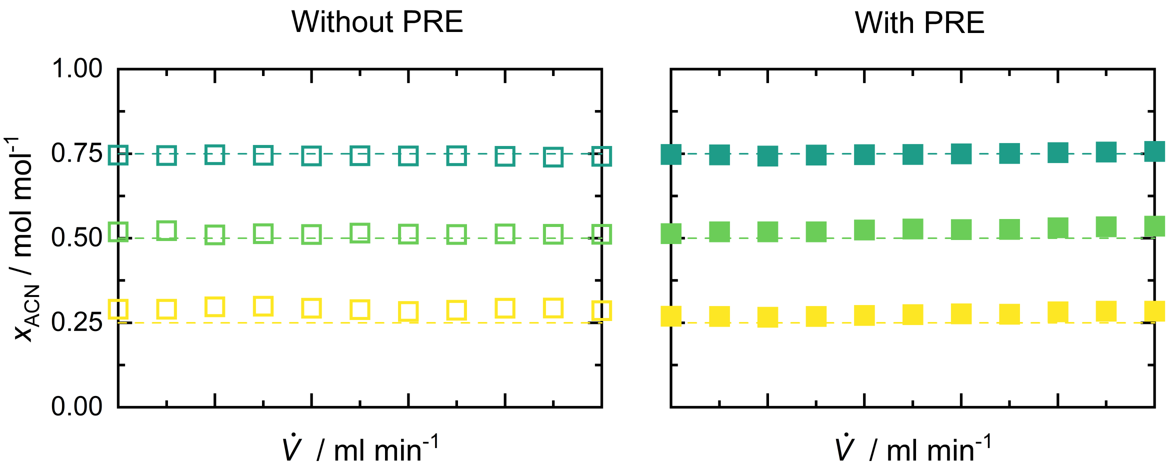

Figure S8: ^{1}H NMR quantification results of acetonitrile (ACN) for system 2 (Mixtures 4-6) based on Eq. (2) and the data shown in Figure S7. Results obtained without PRE (left) and with PRE (right) are shown as a function of flow rate. The dashed lines indicate the gravimetrically determined composition. Error bars are within symbol size.

Figures S9 and S10 show the relative deviation δ_{rel} of the ^{1}H NMR results of ACN and DIOX in system 2 (Mixtures 4-6) as a function of flow rate.

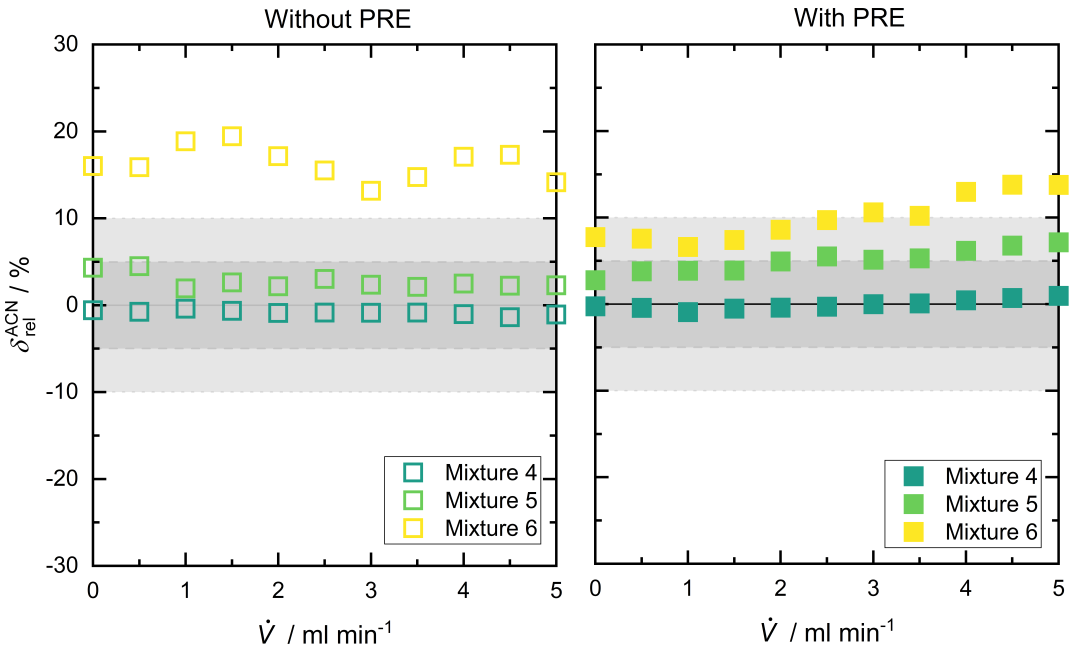

Figure S9: Relative deviations δ_{rel} of the quantification based on ^{1}H NMR results of acetonitrile (ACN) in system 2 (Mixtures 4-6) calculated according to Eq. (S2) and the data shown in Figure S8. δ_{rel} calculated for results without PRE (left) and with PRE (right) are shown as a function of flow rate. The shaded regions represent relative deviations of 5% (dark gray) and 10% (light gray) from the gravimetrically determined composition.

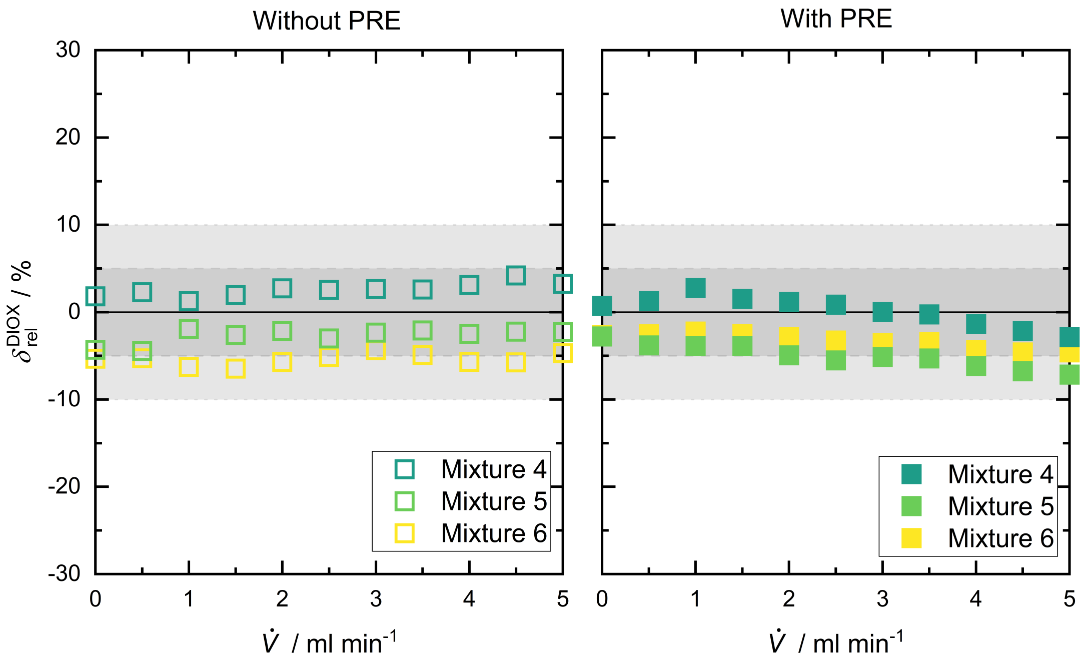

Figure S10: Relative deviations δ_{rel} of the quantification based on ^{1}H NMR results of 1,4-dioxane (DIOX) in system 2 (Mixtures 4-6) calculated according to Eq. (S2) and the data shown in Figure S8. δ_{rel} calculated for results without PRE (left) and with PRE (right) are shown as a function of flow rate. The shaded regions represent relative deviations of 5% (dark gray) and 10% (light gray) from the gravimetrically determined composition.

Tables S11 and S12 give the numerical data of the normalized integrals obtained by ^{1}H NMR of pure ACN and in system 2 (Mixtures 4-6) without and with the PRE agent for the investigated flow rates.

Table S11: Numerical data of the normalized integrals for ^{1}H NMR of pure acetonitrile (ACN) and in system 2 (Mixtures 4-6) without the PRE agent at 42.5 MHz.

Flow rate	$A_{\mathtt{ACN}}$ without PRE			
/ mL min^{-1}	pure	mixture 4	mixture 5	mixture 6
0.0	8.127 (± 0.038)	5.397 (± 0.052)	3.408 (± 0.036)	1.701 (± 0.025)
0.5	8.347 (± 0.007)	5.337 (± 0.017)	3.382 (± 0.016)	1.688 (± 0.075)
1.0	7.800 (± 0.017)	5.060 (± 0.083)	3.086 (± 0.037)	1.676 (± 0.019)
1.5	7.207 (± 0.013)	4.568 (± 0.024)	2.928 (± 0.027)	1.593 (± 0.023)
2.0	6.620 (± 0.013)	4.186 (± 0.019)	2.727 (± 0.025)	1.464 (± 0.008)
2.5	5.907 (± 0.048)	3.838 (± 0.022)	2.556 (± 0.015)	1.333 (± 0.018)
3.0	5.373 (± 0.154)	3.605 (± 0.044)	2.329 (± 0.030)	1.221 (± 0.017)
3.5	5.190 (± 0.016)	3.356 (± 0.036)	2.190 (± 0.007)	1.178 (± 0.013)
4.0	4.873 (± 0.022)	3.117 (± 0.026)	2.093 (± 0.033)	1.150 (± 0.015)
4.5	4.643 (± 0.028)	2.859 (± 0.044)	1.975 (± 0.035)	1.098 (± 0.020)
5.0	4.357 (± 0.022)	2.770 (± 0.054)	1.863 (± 0.025)	1.002 (± 0.012)

Table S12: Numerical data of the normalized integrals for ^{1}H NMR of pure acetonitrile (ACN) and in system 2 (Mixtures 4-6) with the PRE agent at 42.5 MHz.

Flow rate	$A_{\mathtt{ACN}}$ with PRE			
/ mL min^{-1}	pure	mixture 4	mixture 5	mixture 6
0.0	8.403 (± 0.080)	5.570 (± 0.022)	3.424 (± 0.101)	1.798 (± 0.024)
0.5	8.467 (± 0.072)	5.586 (± 0.130)	3.481 (± 0.059)	1.806 (± 0.023)
1.0	8.467 (± 0.072)	5.556 (± 0.019)	3.509 (± 0.036)	1.802 (± 0.040)
1.5	9.290 (± 0.013)	5.636 (± 0.058)	3.508 (± 0.039)	1.765 (± 0.031)
2.0	9.020 (± 0.009)	5.628 (± 0.036)	3.582 (± 0.034)	1.688 (± 0.014)
2.5	8.973 (± 0.033)	5.628 (± 0.026)	3.579 (± 0.060)	1.720 (± 0.017)
3.0	9.327 (± 0.013)	5.615 (± 0.024)	3.495 (± 0.037)	1.670 (± 0.017)
3.5	9.067 (± 0.014)	5.572 (± 0.023)	3.439 (± 0.057)	1.603 (± 0.021)
4.0	8.810 (± 0.009)	5.570 (± 0.028)	3.428 (± 0.054)	1.641 (± 0.027)
4.5	8.433 (± 0.008)	5.470 (± 0.022)	3.377 (± 0.054)	1.644 (± 0.017)
5.0	8.367 (± 0.007)	5.423 (± 0.024)	3.325 (± 0.062)	1.639 (± 0.029)

Tables S13 and S14 give the numerical data of the normalized integrals obtained by ^{1}H NMR of pure DIOX and in system 2 (Mixtures 4-6) without and with the PRE agent for the investigated flow rates.

Table S13: Numerical data of the normalized integrals for ^{1}H NMR of pure 1,4-dioxane (DIOX) and in system 2 (Mixtures 4-6) without the PRE agent at 42.5 MHz.

Flow rate / mL min^{-1}	$A_{\mathtt{DIOX}}$ without PRE			
	pure	mixture 4	mixture 5	mixture 6
0.0	5.151 (± 0.008)	1.842 (± 0.053)	3.126 (± 0.014)	4.164 (± 0.014)
0.5	5.096 (± 0.081)	1.833 (± 0.016)	3.093 (± 0.022)	4.139 (± 0.023)
1.0	5.007 (± 0.048)	1.715 (± 0.070)	2.970 (± 0.030)	3.964 (± 0.022)
1.5	4.788 (± 0.034)	1.562 (± 0.017)	2.778 (± 0.051)	3.742 (± 0.030)
2.0	4.523 (± 0.026)	1.447 (± 0.024)	2.612 (± 0.072)	3.534 (± 0.047)
2.5	4.294 (± 0.032)	1.323 (± 0.023)	2.406 (± 0.026)	3.285 (± 0.018)
3.0	4.057 (± 0.028)	1.245 (± 0.029)	2.223 (± 0.058)	3.096 (± 0.034)
3.5	3.859 (± 0.031)	1.158 (± 0.044)	2.099 (± 0.021)	2.929 (± 0.045)
4.0	3.664 (± 0.051)	1.083 (± 0.038)	1.992 (± 0.030)	2.778 (± 0.022)
4.5	3.507 (± 0.044)	1.007 (± 0.048)	1.889 (± 0.112)	2.645 (± 0.019)
5.0	3.371 (± 0.040)	0.964 (± 0.046)	1.780 (± 0.053)	2.509 (± 0.021)

Table S14: Numerical data of the normalized integrals for ^{1}H NMR of pure 1,4-dioxane (DIOX) and in system 2 (Mixtures 4-6) with the PRE agent at 42.5 MHz.

Flow rate / mL min^{-1}	$A_{\mathtt{DIOX}}$ with PRE			
	pure	mixture 4	mixture 5	mixture 6
0.0	5.171 (± 0.061)	1.875 (± 0.016)	3.239 (± 0.066)	4.306 (± 0.018)
0.5	5.196 (± 0.036)	1.894 (± 0.199)	3.226 (± 0.050)	4.302 (± 0.104)
1.0	5.229 (± 0.031)	1.921 (± 0.037)	3.249 (± 0.026)	4.322 (± 0.040)
1.5	5.224 (± 0.027)	1.918 (± 0.042)	3.246 (± 0.036)	4.332 (± 0.014)
2.0	5.164 (± 0.022)	1.906 (± 0.039)	3.246 (± 0.034)	4.305 (± 0.024)
2.5	5.071 (± 0.045)	1.898 (± 0.021)	3.206 (± 0.057)	4.248 (± 0.011)
3.0	4.951 (± 0.047)	1.872 (± 0.027)	3.153 (± 0.023)	4.185 (± 0.013)
3.5	4.821 (± 0.046)	1.851 (± 0.017)	3.093 (± 0.052)	4.108 (± 0.022)
4.0	4.689 (± 0.040)	1.824 (± 0.038)	3.030 (± 0.032)	3.987 (± 0.023)
4.5	4.551 (± 0.028)	1.771 (± 0.023)	2.950 (± 0.066)	3.898 (± 0.015)
5.0	4.414 (± 0.017)	1.740 (± 0.025)	2.881 (± 0.075)	3.823 (± 0.033)

^{13}C NMR of System 2

Figure S11 shows the relative polarization S/S_0 of ^{13}C of ACN (left) and DIOX (right) for the pure solvents and system 2 (Mixtures 4-6; see Table 2 main part) as a function of flow rate. All spectra were recorded using PENDANT with 8 scans. Results obtained with PRE (filled symbols) are compared to measurements without PRE (open symbols) and to the

static reference experiments (dashed line). Here, the same trends are present for system 1. The polarization of ACN and DIOX is enhanced with PRE and is almost constant for all investigated flow rates and mixtures.

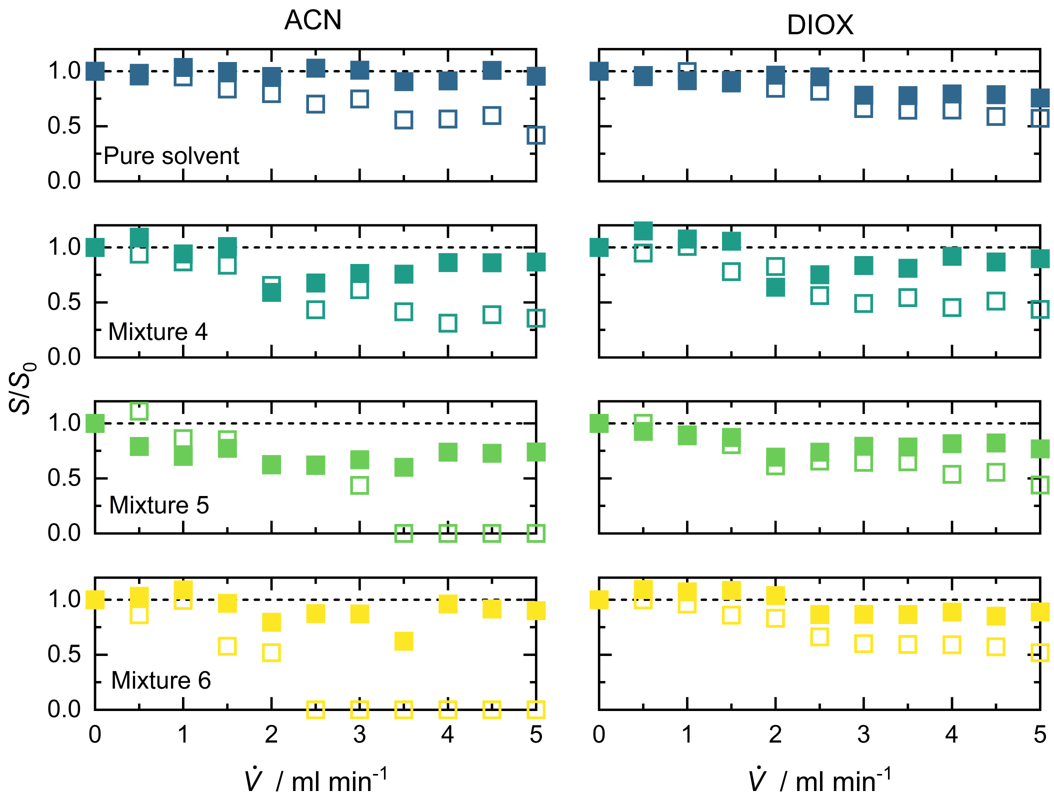

Figure S11: ^{13}C NMR relative polarization S/S_0 of acetonitrile (ACN, left) and 1,4-dioxane (DIOX, right) for the pure solvents and system 2 (Mixtures 4-6) as a function of flow rate. Spectra were recorded using PENDANT with 8 scans. Data obtained with PRE are shown as filled symbols, data obtained without PRE as open symbols; dashed lines indicate the static reference experiments. Error bars are within symbol size.

Figures S12 and S13 show the relative deviation δ_{rel} of the ^{13}C NMR results, recorded with PENDANT, of ACN and DIOX in system 2 (Mixtures 4-6) as a function of flow rate. With the PRE agent, the relative deviations of the obtained mole fractions of ACN and DIOX for Mixtures 4-6 are significantly reduced.

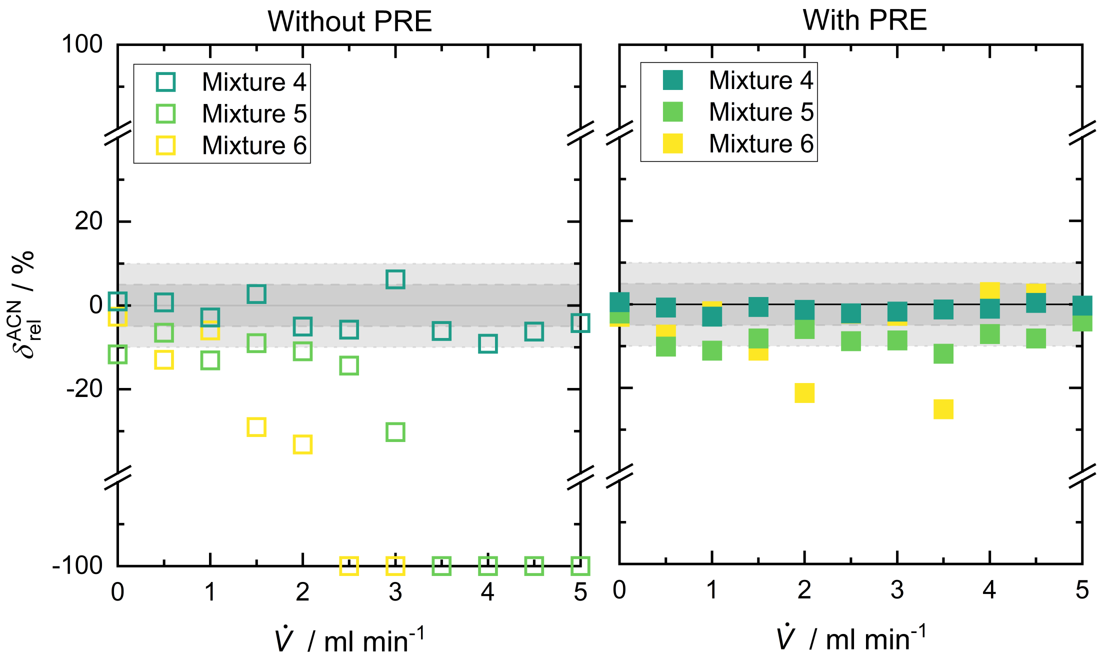

Figure S12: Relative deviations δ_{rel} of the quantification based on ^{13}C NMR results, recorded with PENDANT, of acetonitrile (ACN) in system 2 (Mixtures 4-6) calculated according to Eq. (S2) of the data shown in Figure 6. δ_{rel} calculated for results without PRE (left) and with PRE (right) are shown as a function of flow rate. The shaded regions represent relative deviations of 5% (dark gray) and 10% (light gray) from the gravimetrically determined composition.

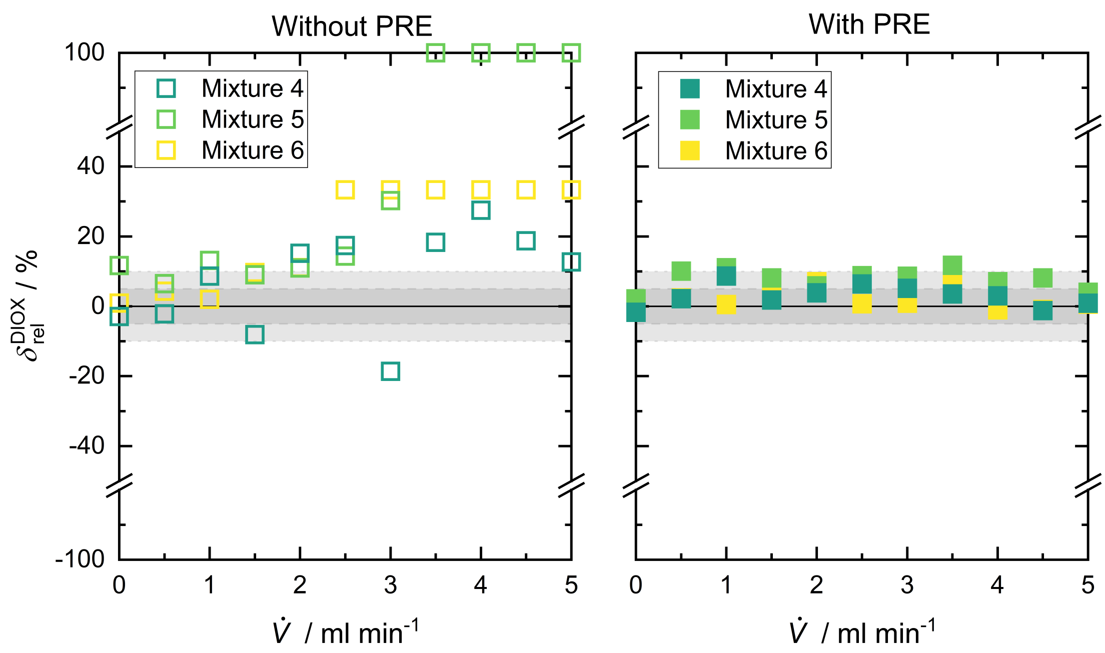

Figure S13: Relative deviations δ_{rel} of the quantification based on ^{13}C NMR results, recorded with PENDANT, of 1,4-dioxane (DIOX) in system 2 (Mixtures 4-6) calculated according to Eq. (S2) of the data shown in Figure 6. δ_{rel} calculated for results without PRE (left) and with PRE (right) are shown as a function of flow rate. The shaded regions represent relative deviations of 5% (dark gray) and 10% (light gray) from the gravimetrically determined composition.

Tables S15 and S16 give the numerical data of the normalized integrals obtained by ^{13}C NMR, recorded with PENDANT, of pure ACN and in system 2 (Mixtures 4-6) without and with the PRE agent for the investigated flow rates.

Table S15: Numerical data of the normalized integrals for ^{13}C NMR, recorded with PENDANT, of pure acetonitrile (ACN) and in system 2 (Mixtures 4-6) without the PRE agent at 42.5 MHz.

Flow rate	$A_{ACN} \times 10^{-3}$ without PRE			
/ mL min^{-1}	pure	mixture 4	mixture 5	mixture 6
0.0	6.627 (± 0.740)	4.284 (± 0.545)	2.147 (± 0.272)	1.190 (± 0.236)
0.5	6.504 (± 0.502)	4.018 (± 0.537)	2.382 (± 0.631)	1.027 (± 0.264)
1.0	6.858 (± 0.782)	3.714 (± 0.163)	1.847 (± 0.291)	1.035 (± 0.637)
1.5	6.607 (± 0.991)	3.590 (± 0.400)	1.825 (± 0.321)	0.686 (± 0.465)
2.0	6.317 (± 0.766)	2.802 (± 0.316)	1.336 (± 0.119)	0.617 (± 0.446)
2.5	6.816 (± 0.967)	1.857 (± 0.467)	1.335 (± 0.298)	0
3.0	6.694 (± 0.847)	2.632 (± 0.721)	0.938 (± 0.287)	0
3.5	6.001 (± 0.684)	1.773 (± 0.308)	0	0
4.0	6.052 (± 1.510)	1.329 (± 0.221)	0	0
4.5	6.677 (± 0.854)	1.661 (± 0.191)	0	0
5.0	6.328 (± 0.981)	1.528 (± 0.362)	0	0

Table S16: Numerical data of the normalized integrals for ^{13}C NMR, recorded with PENDANT, of pure acetonitrile (ACN) and in system 2 (Mixtures 4-6) with the PRE agent at 42.5 MHz.

Flow rate	$A_{ACN} \times 10^{-3}$ with PRE			
/ mL min^{-1}	pure	mixture 4	mixture 5	mixture 6
0.0	6.627 (± 0.740)	3.989 (± 0.555)	3.083 (± 0.703)	1.258 (± 0.186)
0.5	6.504 (± 0.502)	4.360 (± 0.531)	2.439 (± 0.247)	1.297 (± 0.314)
1.0	6.858 (± 0.782)	3.751 (± 0.289)	2.278 (± 0.213)	1.372 (± 0.221)
1.5	6.607 (± 0.991)	4.022 (± 0.379)	2.384 (± 0.397)	1.218 (± 0.311)
2.0	6.317 (± 0.766)	2.364 (± 0.464)	2.037 (± 0.302)	1.001 (± 0.228)
2.5	6.816 (± 0.967)	2.699 (± 0.239)	1.993 (± 0.347)	1.100 (± 0.393)
3.0	6.694 (± 0.847)	3.047 (± 0.471)	2.066 (± 0.555)	1.096 (± 0.169)
3.5	6.001 (± 0.684)	3.017 (± 0.175)	1.967 (± 0.287)	0.784 (± 0.107)
4.0	6.052 (± 1.510)	3.444 (± 0.274)	2.276 (± 0.764)	1.209 (± 0.184)
4.5	6.677 (± 0.854)	3.434 (± 0.505)	2.246 (± 0.328)	1.152 (± 0.469)
5.0	6.328 (± 0.981)	3.459 (± 0.629)	2.280 (± 0.323)	1.134 (± 0.385)

Tables S17 and S18 give the numerical data of the normalized integrals obtained by ^{13}C NMR, recorded with PENDANT, of pure DIOX and in system 2 (Mixtures 4-6) without and with the PRE agent for the investigated flow rates.

Table S17: Numerical data of the normalized integrals for ^{13}C NMR, recorded with PENDANT, of pure 1,4-dioxane (DIOX) and in system 2 (Mixtures 4-6) without the PRE agent at 42.5 MHz.

Flow rate / mL min^{-1}	$A_{\mathtt{DIOX}} \times 10^{-3}$ without PRE			
	pure	mixture 4	mixture 5	mixture 6
0.0	4.076 (± 0.079)	1.262 (± 0.037)	2.108 (± 0.133)	2.890 (± 0.097)
0.5	3.861 (± 0.123)	1.131 (± 0.117)	2.048 (± 0.102)	2.873 (± 0.070)
1.0	4.052 (± 0.131)	1.204 (± 0.135)	1.812 (± 0.131)	2.619 (± 0.182)
1.5	3.636 (± 0.149)	0.992 (± 0.060)	1.766 (± 0.126)	2.420 (± 0.147)
2.0	3.469 (± 0.174)	0.982 (± 0.096)	1.226 (± 0.118)	2.463 (± 0.117)
2.5	3.327 (± 0.146)	0.690 (± 0.106)	1.269 (± 0.176)	1.903 (± 0.106)
3.0	2.641 (± 0.162)	0.587 (± 0.098)	1.347 (± 0.064)	1.716 (± 0.006)
3.5	2.651 (± 0.120)	0.666 (± 0.065)	1.316 (± 0.150)	1.706 (± 0.153)
4.0	2.644 (± 0.059)	0.520 (± 0.027)	1.061 (± 0.184)	1.821 (± 0.079)
4.5	2.391 (± 0.119)	0.627 (± 0.100)	1.151 (± 0.113)	1.645 (± 0.037)
5.0	2.377 (± 0.115)	0.518 (± 0.059)	1.002 (± 0.119)	1.479 (± 0.145)

Table S18: Numerical data of the normalized integrals for ^{13}C NMR, recorded with PENDANT, of pure 1,4-dioxane (DIOX) and in system 2 (Mixtures 4-6) with the PRE agent at 42.5 MHz.

Flow rate / mL min^{-1}	$A_{\mathtt{DIOX}} \times 10^{-3}$ with PRE			
	pure	mixture 4	mixture 5	mixture 6
0.0	4.140 (± 0.181)	1.146 (± 0.198)	2.639 (± 0.077)	3.178 (± 0.016)
0.5	3.980 (± 0.066)	1.338 (± 0.068)	2.318 (± 0.092)	3.342 (± 0.117)
1.0	3.787 (± 0.082)	1.309 (± 0.065)	2.240 (± 0.093)	3.271 (± 0.063)
1.5	3.790 (± 0.025)	1.312 (± 0.098)	2.179 (± 0.061)	3.361 (± 0.077)
2.0	3.990 (± 0.046)	0.742 (± 0.116)	1.735 (± 0.113)	3.216 (± 0.064)
2.5	3.928 (± 0.114)	0.878 (± 0.080)	1.847 (± 0.059)	2.643 (± 0.066)
3.0	3.239 (± 0.186)	0.965 (± 0.062)	1.952 (± 0.232)	2.756 (± 0.063)
3.5	3.222 (± 0.181)	1.038 (± 0.148)	1.962 (± 0.089)	2.767 (± 0.134)
4.0	3.287 (± 0.128)	1.060 (± 0.113)	2.008 (± 0.114)	2.619 (± 0.089)
4.5	3.257 (± 0.118)	1.008 (± 0.085)	2.054 (± 0.050)	2.677 (± 0.224)
5.0	3.137 (± 0.094)	1.087 (± 0.094)	1.920 (± 0.173)	2.794 (± 0.161)

5 System 3: 1,4-Dioxane + Acetonitrile + Ethanol

^{13}C NMR of System 3

Figures S14, S15 and S16 show the relative deviations δ_{rel} of the ^{13}C NMR results, recorded with PENDANT, of ACN, EOH and DIOX in system 3 (Mixtures 7-10) as a function of flow rate. With the PRE agent, the relative deviations of the obtained mole fractions of ACN, EOH and DIOX for Mixtures 7-10 are reduced.

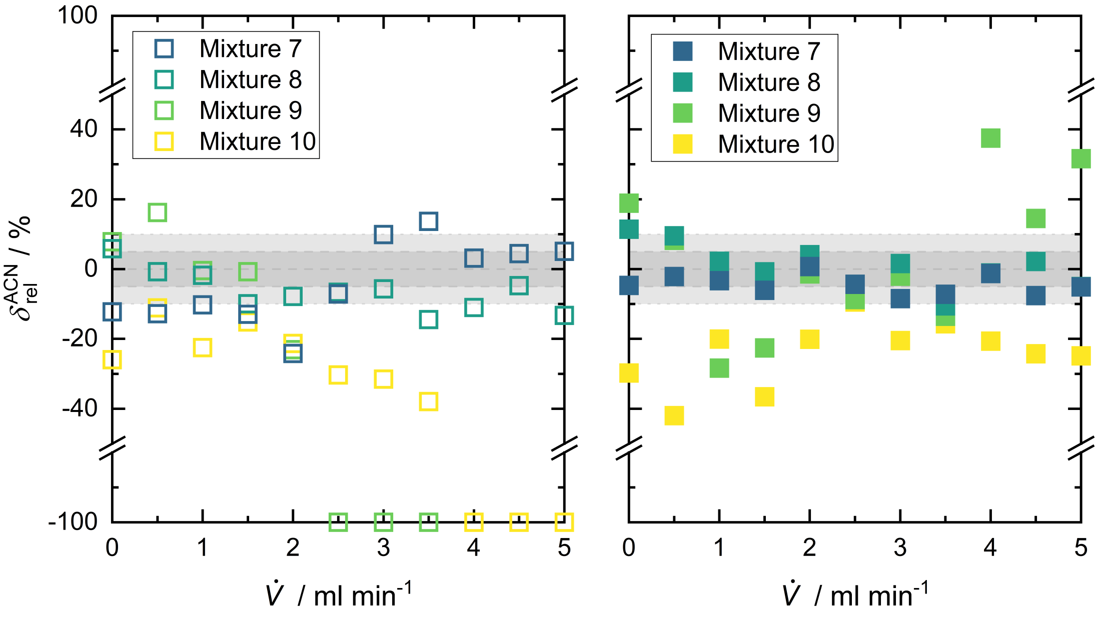

Figure S14: Relative deviations δ_{rel} of the quantification based on ^{13}C NMR results, recorded with PENDANT, of acetonitrile (ACN) in system 3 (Mixtures 7-10) calculated according to Eq. (S2) of the data shown in Figure 7. δ_{rel} calculated for results without PRE (left) and with PRE (right) are shown as a function of flow rate. The shaded regions represent relative deviations of 5% (dark gray) and 10% (light gray) from the gravimetrically determined composition.

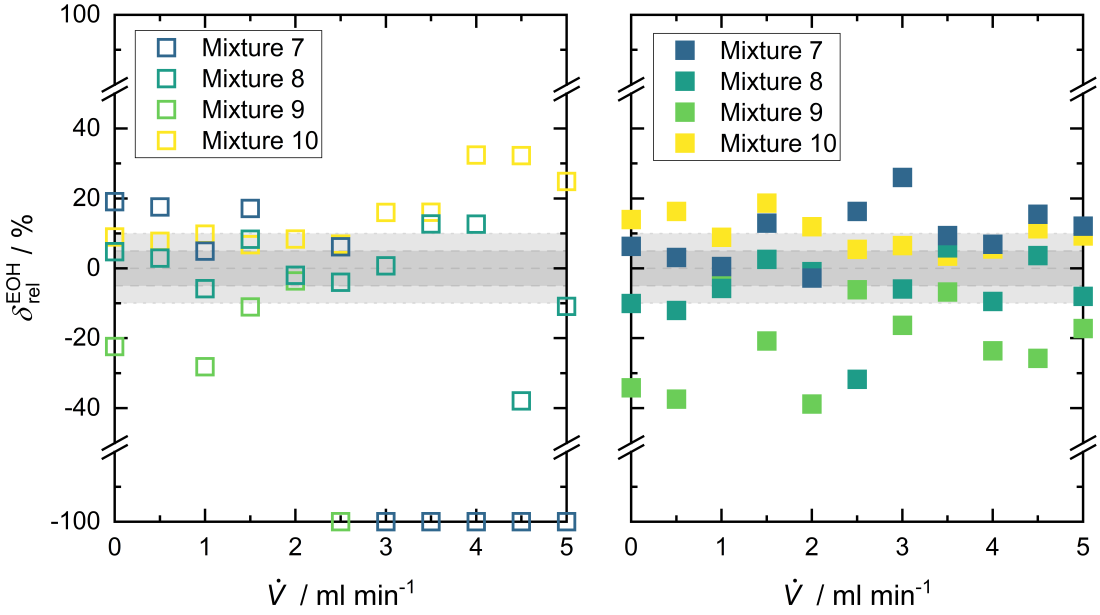

Figure S15: Relative deviations δ_{rel} of the quantification based on ^{13}C NMR results, recorded with PENDANT, of ethanol (EOH) in system 3 (Mixtures 7-10) calculated according to Eq. (S2) of the data shown in Figure 7. δ_{rel} calculated for results without PRE (left) and with PRE (right) are shown as a function of flow rate. The shaded regions represent relative deviations of 5% (dark gray) and 10% (light gray) from the gravimetrically determined composition.

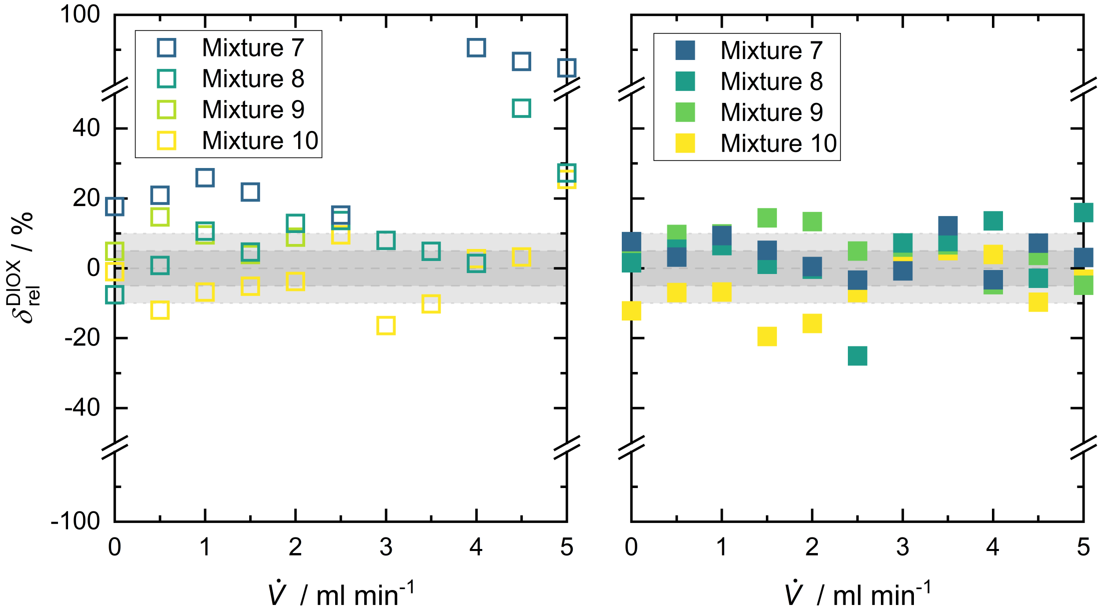

Figure S16: Relative deviations δ_{rel} of the quantification based on ^{13}C NMR results, recorded with PENDANT, of 1,4-dioxane (DIOX) in system 3 (Mixtures 7-10) calculated according to Eq. (S2) and the data shown in Figure 7. δ_{rel} calculated for results without PRE (left) and with PRE (right) are shown as a function of flow rate. The shaded regions represent relative deviations of 5% (dark gray) and 10% (light gray) from the gravimetrically determined composition.

Tables S19 and S20 give the numerical data of the normalized integrals obtained by ^{13}C NMR, recorded with PENDANT, of ACN in system 3 (Mixtures 7-10) without and with the PRE agent for the investigated flow rates.

Table S19: Numerical data of the normalized integrals for ^{13}C NMR, recorded with PENDANT, of acetonitrile (ACN) in system 3 (Mixtures 7-10) without the PRE agent at 42.5 MHz.

Flow rate / mL min^{-1}	$A_{ACN} \times 10^{-3}$ without PRE			
	mixture 7	mixture 8	mixture 9	mixture 10
0.0	3.497 (± 0.387)	2.067 (± 0.308)	1.135 (± 0.325)	0.960 (± 0.274)
0.5	3.178 (± 0.655)	1.886 (± 0.270)	1.106 (± 0.407)	1.285 (± 0.119)
1.0	3.414 (± 0.285)	1.603 (± 0.387)	0.961 (± 0.394)	1.103 (± 0.148)
1.5	2.539 (± 0.434)	1.388 (± 0.271)	0.903 (± 0.160)	1.041 (± 0.284)
2.0	2.166 (± 0.571)	1.230 (± 0.335)	0.626 (± 0.289)	0.842 (± 0.188)
2.5	1.985 (± 0.564)	0.966 (± 0.361)	0	0.565 (± 0.480)
3.0	1.556 (± 0.451)	0.947 (± 0.366)	0	0.569 (± 0.262)
3.5	1.822 (± 0.587)	0.872 (± 0.215)	0	0.541 (± 0.107)
4.0	1.568 (± 0.648)	0.965 (± 0.161)	0	0
4.5	1.368 (± 0.265)	0.712 (± 0.414)	0	0
5.0	1.346 (± 0.177)	0.707 (± 0.312)	0	0

Table S20: Numerical data of the normalized integrals for ^{13}C NMR, recorded with PENDANT, of acetonitrile (ACN) in system 3 (Mixtures 7-10) with the PRE agent at 42.5 MHz.

Flow rate / mL min^{-1}	$A_{ACN} \times 10^{-3}$ with PRE			
	mixture 7	mixture 8	mixture 9	mixture 10
0.0	3.591 (± 0.246)	1.936 (± 0.317)	1.188 (± 0.285)	0.937 (± 0.209)
0.5	3.654 (± 0.294)	1.865 (± 0.335)	1.060 (± 0.158)	0.812 (± 0.188)
1.0	3.726 (± 0.294)	1.797 (± 0.250)	0.693 (± 0.243)	1.091 (± 0.156)
1.5	3.499 (± 0.477)	1.646 (± 0.457)	0.694 (± 0.225)	0.829 (± 0.558)
2.0	3.644 (± 0.351)	1.399 (± 0.455)	0.892 (± 0.096)	1.008 (± 0.274)
2.5	3.069 (± 0.310)	1.514 (± 0.196)	0.742 (± 0.194)	0.926 (± 0.511)
3.0	3.055 (± 0.056)	1.413 (± 0.105)	0.809 (± 0.399)	0.869 (± 0.378)
3.5	3.016 (± 0.334)	1.262 (± 0.562)	0.701 (± 0.191)	0.928 (± 0.296)
4.0	3.293 (± 0.239)	1.386 (± 0.472)	1.249 (± 0.136)	0.877 (± 0.194)
4.5	2.992 (± 0.462)	1.354 (± 0.621)	0.899 (± 0.152)	0.889 (± 0.254)
5.0	3.160 (± 0.409)	1.311 (± 0.134)	1.183 (± 0.317)	0.907 (± 0.154)

Tables S21 and S22 give the numerical data of the normalized integrals obtained by ^{13}C NMR, recorded with PENDANT, of EOH in system 3 (Mixtures 7-10) without and with the PRE agent for the investigated flow rates.

Table S21: Numerical data of the normalized integrals for ^{13}C NMR, recorded with PENDANT, of ethanol (EOH) in system 3 (Mixtures 7-10) without the PRE agent at 42.5 MHz.

Flow rate / mL min^{-1}	$A_{\texttt{EOH}} \times 10^{-3}$ without PRE			
	mixture 7	mixture 8	mixture 9	mixture 10
0.0	1.188 (± 0.171)	1.926 (± 0.250)	0.961 (± 0.163)	2.814 (± 0.279)
0.5	1.073 (± 0.337)	1.840 (± 0.294)	0.446 (± 0.126)	3.100 (± 0.241)
1.0	0.999 (± 0.173)	1.445 (± 0.480)	0.816 (± 0.103)	3.110 (± 0.161)
1.5	0.856 (± 0.228)	1.570 (± 0.442)	0.953 (± 0.379)	2.610 (± 0.444)
2.0	0.980 (± 0.371)	1.231 (± 0.348)	0.924 (± 0.285)	2.308 (± 0.383)
2.5	0.568 (± 0.361)	0.934 (± 0.288)	0	1.726 (± 0.296)
3.0	0	0.951 (± 0.325)	0	1.919 (± 0.459)
3.5	0	1.081 (± 0.191)	0	2.015 (± 0.206)
4.0	0	1.150 (± 0.461)	0	1.743 (± 0.471)
4.5	0	0.436 (± 0.244)	0	1.565 (± 0.340)
5.0	0	0.683 (± 0.319)	0	1.309 (± 0.336)

Table S22: Numerical data of the normalized integrals for ^{13}C NMR, recorded with PENDANT, of ethanol (EOH) in system 3 (Mixtures 7-10) with the PRE agent at 42.5 MHz.

Flow rate / mL min^{-1}	$A_{\texttt{EOH}} \times 10^{-3}$ with PRE			
	mixture 7	mixture 8	mixture 9	mixture 10
0.0	1.003 (± 0.216)	1.471 (± 0.112)	0.774 (± 0.255)	3.027 (± 0.174)
0.5	0.964 (± 0.333)	1.409 (± 0.250)	0.721 (± 0.428)	3.239 (± 0.418)
1.0	0.970 (± 0.651)	1.559 (± 0.230)	1.123 (± 0.313)	2.958 (± 0.412)
1.5	1.053 (± 0.229)	1.601 (± 0.238)	0.837 (± 0.375)	3.086 (± 0.365)
2.0	0.881 (± 0.098)	1.252 (± 0.286)	0.651 (± 0.264)	2.813 (± 0.573)
2.5	0.934 (± 0.386)	1.177 (± 0.252)	0.898 (± 0.145)	2.146 (± 0.262)
3.0	1.053 (± 0.052)	1.231 (± 0.221)	0.814 (± 0.258)	2.318 (± 0.493)
3.5	0.890 (± 0.201)	1.405 (± 0.458)	0.889 (± 0.306)	2.268 (± 0.122)
4.0	0.892 (± 0.307)	1.194 (± 0.221)	0.817 (± 0.238)	2.323 (± 0.415)
4.5	0.936 (± 0.278)	1.292 (± 0.137)	0.686 (± 0.200)	2.603 (± 0.198)
5.0	0.935 (± 0.210)	1.195 (± 0.202)	0.876 (± 0.203)	2.628 (± 0.247)

Tables S23 and S24 give the numerical data of the normalized integrals obtained by ^{13}C NMR, recorded with PENDANT, of DIOX in system 3 (Mixtures 7-10) without and with the PRE agent for the investigated flow rates.

Table S23: Numerical data of the normalized integrals for ^{13}C NMR, recorded with PENDANT, of 1,4-dioxane (DIOX) in system 3 (Mixtures 7-10) without the PRE agent at 42.5 MHz.

Flow rate	$A_{\mathtt{DIOX}} \times 10^{-3}$ without PRE			
/ mL min^{-1}	mixture 7	mixture 8	mixture 9	mixture 10
0.0	4.286 (± 0.446)	6.745 (± 0.308)	11.58 (± 0.174)	4.047 (± 0.293)
0.5	4.029 (± 0.341)	7.153 (± 0.473)	11.46 (± 0.808)	4.008 (± 0.373)
1.0	4.378 (± 0.230)	6.743 (± 0.455)	11.10 (± 0.749)	4.178 (± 0.305)
1.5	3.250 (± 0.542)	6.021 (± 0.343)	9.936 (± 0.401)	3.669 (± 0.367)
2.0	3.542 (± 0.833)	5.623 (± 0.475)	9.311 (± 0.408)	3.243 (± 0.179)
2.5	2.252 (± 0.447)	4.390 (± 0.430)	7.256 (± 0.315)	2.800 (± 0.556)
3.0	2.205 (± 0.540)	4.050 (± 0.308)	7.082 (± 0.198)	2.189 (± 0.407)
3.5	2.329 (± 0.501)	3.992 (± 0.314)	7.036 (± 0.350)	2.468 (± 0.263)
4.0	2.649 (± 0.511)	4.109 (± 0.468)	7.063 (± 0.366)	2.140 (± 0.383)
4.5	2.235 (± 0.507)	4.070 (± 0.569)	6.142 (± 0.517)	1.933 (± 0.250)
5.0	2.165 (± 0.320)	3.873 (± 0.440)	6.511 (± 0.446)	2.080 (± 0.425)

Table S24: Numerical data of the normalized integrals for ^{13}C NMR, recorded with PENDANT, of 1,4-dioxane (DIOX) in system 3 (Mixtures 7-10) with the PRE agent at 42.5 MHz.

Flow rate	$A_{\mathtt{DIOX}} \times 10^{-3}$ with PRE			
/ mL min^{-1}	mixture 7	mixture 8	mixture 9	mixture 10
0.0	3.707 (± 0.364)	6.594 (± 0.373)	11.03 (± 0.546)	3.693 (± 0.355)
0.5	3.522 (± 0.528)	6.708 (± 0.201)	11.28 (± 0.773)	4.102 (± 0.436)
1.0	3.853 (± 0.070)	6.990 (± 0.218)	11.16 (± 0.545)	4.007 (± 0.413)
1.5	3.580 (± 0.182)	6.265 (± 0.362)	10.78 (± 0.656)	3.313 (± 0.398)
2.0	3.323 (± 0.149)	5.005 (± 0.228)	10.76 (± 0.605)	3.349 (± 0.260)
2.5	2.834 (± 0.394)	5.024 (± 0.295)	8.957 (± 0.295)	2.997 (± 0.243)
3.0	3.027 (± 0.290)	5.572 (± 0.265)	9.198 (± 0.599)	3.474 (± 0.187)
3.5	3.333 (± 0.107)	5.671 (± 0.682)	9.077 (± 0.375)	3.642 (± 0.596)
4.0	2.948 (± 0.436)	5.947 (± 0.153)	9.094 (± 0.245)	3.622 (± 0.485)
4.5	3.174 (± 0.397)	4.809 (± 1.566)	8.546 (± 0.433)	3.342 (± 0.556)
5.0	3.139 (± 0.388)	5.978 (± 0.406)	8.984 (± 0.480)	3.687 (± 0.397)

References

(1) Kircher, R.; Mross, S.; Hasse, H.; Münnemann, K. Quantitative Analysis in Continuous-Flow 1H Benchtop NMR Spectroscopy by Paramagnetic Relaxation Enhancement. *Applied Magnetic Resonance* **2023**, *54*, 1555–1569, DOI: `10.1007/s00723-023-01626-8`.
